# Multi-Dot/Icm effector mutants reveal distinct host responses in *Legionella* infected macrophages

**DOI:** 10.1371/journal.ppat.1014083

**Published:** 2026-10-22

**Authors:** Raissa Wibawa, Pengfei Li, Linden J. Gearing, Sacha Pidot, Yuliia Faidiuk, Wilson Wong, Garrett Z. Ng, Kathleen McCaffrey, Paul J. Hertzog, Shivani Pasricha, Elizabeth L. Hartland

**Affiliations:** 1 Centre for Innate Immunity and Infectious Diseases, Hudson Institute of Medical Research, Clayton, Australia; 2 Department of Microbiology and Immunology, University of Melbourne at the Peter Doherty Institute for Infection and Immunity, Melbourne, Australia; 3 Department of Molecular and Translational Science, Monash University, Clayton, Australia; 4 Department of Infectious Diseases, University of Melbourne at the Peter Doherty Institute for Infection and Immunity, Melbourne, Australia; The First Hospital of Jilin University, CHINA

## Abstract

*Legionella pneumophila* is the causative agent of Legionnaires’ disease, an accidental and severe form of pneumonia. The replication of *L. pneumophila* within alveolar macrophages requires the Dot/Icm type IV secretion system that translocates more than 330 bacterial effector proteins into the infected host cell. Many Dot/Icm effectors possess eukaryotic-like domains and manipulate host processes by functional mimicry. However, only a minority of effectors have been fully characterized. Redundancy among effector protein activities and host targets has complicated functional characterization of individual effector proteins. To explore the collective impact of Dot/Icm effectors on host cell processes, we created a library of 14 *L. pneumophila* genomic mutant strains lacking effector-rich regions (a total of 85 effector genes deleted) and used the multi-deletion mutants to interrogate host-pathogen interactions. While none of the mutants exhibited a defect in intracellular replication in THP-1 macrophages, several were attenuated in amoebae, including a mutant lacking a predicted amino acid transporter. In THP-1 cells, we observed that wild-type *L. pneumophila* 130b triggered a strong inflammatory and cellular stress transcriptional response in macrophages, whereas six mutants displayed reduced interferon signalling and one mutant displayed a marked decrease in the global expression of host heat shock response (HSR) genes. Through the creation of further sub-deletions, we found that the Dot/Icm effector kinase LegK4 was responsible for induction of the host HSR via the canonical transcription factor heat shock factor 1 (HSF1). Infection of THP-1 macrophages deficient in *HSF1* revealed a potential role for the host HSR in the early stages of *L. pneumophila* infection. Together, these results reinforce the utility of multi-gene deletions to identify Dot/Icm effector phenotypes and the importance of the host HSR to *L. pneumophila* infection.

## Author summary

The bacterial pathogen *Legionella pneumophila* modulates host cell processes to replicate in human macrophages. Intracellular replication requires the *Legionella* Dot/Icm type IV secretion system that translocates more than 330 effector proteins into the infected macrophage that collectively enable intracellular replication. Here, we created a library of multi-effector deletion mutants to study the role of Dot/Icm effectors in the modulation of macrophage transcriptional responses during infection. While none of the mutants exhibited a defect in intracellular replication in THP-1 macrophages, several were attenuated in amoebae. Wild type *L. pneumophila* induced a strong host inflammatory and cell stress response in macrophages while some mutants produced a weaker response, despite similar levels of bacterial replication. We found that the effector protein LegK4 induced a global heat shock transcriptional response that was dependent on the host heat shock factor 1 (HSF1). Infection of HSF1-deficient macrophages with *L. pneumophila* suggested a potential role for HSF1 in the early stages of *L. pneumophila* infection.

## Introduction

Legionnaires’ disease is a severe form of pneumonia with an increasing incidence worldwide [1,2]. Approximately 90% of cases are caused by *Legionella pneumophila* [3]. *L. pneumophila* infects human alveolar macrophages and establishes an intracellular replicative niche termed the *Legionella*-containing vacuole (LCV). LCV formation requires the bacterial Dot/Icm type IV secretion system (T4SS) that translocates more than 330 effector proteins into the infected cells [4–6].

Through unique interaction or modification of host cell components, Dot/Icm effectors modulate a diverse range of host processes, including ER-Golgi trafficking [7–13], lipid and phosphoinositide metabolism [14–18], ubiquitination homeostasis [19–22], and the transcription and translation machinery [23–26]. Some Dot/Icm effector proteins have functional domains and eukaryotic-like motifs [4,27] that can be used to modify host targets. For example, the effector proteins LegK1–4 and LegK7 possess kinase domains that can phosphorylate various host proteins in macrophages. LegK1 phosphorylates host IkB to activate the NF-kB response [28], while LegK2 inhibits actin polymerization by phosphorylating host ARP2/3 complex to remodel the host cytoskeleton and evade the late endocytic pathway [29]. LegK3 targets host caspases to prevent host cell death [30], and LegK4 phosphorylates host HSP70 to inhibit host protein synthesis [24]. Lastly, LegK7 activates the Hippo pathway to alter host gene expression by phosphorylating host Mps-one-binder 1 (MOB1) [31–33].

Many Dot/Icm effectors modify host proteins in a non-canonical manner. For example, SidE family effectors modify host proteins with ubiquitin in an E1- and E2-independent manner by creating an ADP ribosylated-ubiquitin intermediate (ADPR-Ub) using a mono-ADP-ribosyltransferase domain, and subsequently attaching ADPR-Ub to the host protein target using a phosphodiesterase domain [19,21,34]. This modification can be reversed by the deubiquitinase effectors DupA and DupB that then produce a phosphoribosyl-ubiquitin (PR-Ub) by-product [35]. Cellular levels of both PR-Ub and ADPR-Ub are managed by two additional Dot/Icm effectors, the AMPylase LnaB which converts PR-Ub into ADPR-Ub, which is further processed to ADP-ribose and ubiquitin by the ADP-ribosylhydrolase effector MavL [22,36]

Although the Dot/Icm system encodes many effectors with the potential to reveal novel biochemical activities and mechanisms of host manipulation, their sheer number- together with functional redundancy makes systematic characterization difficult. Effector phenotypes have been screened using a range of different approaches, including transfection of single tagged effectors into the human epithelial cell line HEK293T [28,37] yeast-based mutagenesis screening [38], high-throughput transposon mutagenesis [39], a combination of transposon mutagenesis with siRNA silencing [40], CRISPR interference (CRISPRi) [41,42] and the construction of genomic deletion mutants lacking multiple effector genes in *L. pneumophila* strain Philadelphia-1 [43]. These latter mutants have since been used highly effectively to identify the roles of several Dot/Icm effectors during infection [30,44,45].

Using a similar approach, here we generated a library of 14 *L. pneumophila* strain 130b mutants lacking regions enriched in Dot/Icm effector genes. To assess how these multi-gene deletions affected intracellular replication and host responses in macrophages (THP-1 cells) and the environmental host *Acanthamoeba castellanii*, we performed intracellular replication assays and global transcriptomic analysis to identify host pathways influenced by *L. pneumophila* effector activity. Several of our mutants resulted in weakened interferon signalling by macrophages, with one mutant identified as defective for induction of the host heat shock response (HSR). Further experiments showed that LegK4, which phosphorylates and inactivates HSP70 [24], thereby inducing proteotoxic stress, drove a global heat shock transcriptional response through the host transcription factor, heat shock factor 1 (HSF1). Deficiency in HSF1 influenced bacterial uptake into macrophages and subsequent CFU at 24 h, suggesting that triggering the host heat shock response aids *L. pneumophila* infection.

## Results

### Deletion of Dot/Icm effector gene clusters in *L. pneumophila* strain 130b

Based on previously reported studies, a list of Dot/Icm effector-encoding genes was created and cross-referenced with the *L. pneumophila* 130b genome [4,26,46] (S1 Table). Genes that encode secretion systems in *L. pneumophila* 130b strain [46] were also indicated in S1 Table. Effector-encoding genes were grouped based on their proximity and to maximize the number of effector-encoding genes present while minimizing the number of non-effector genes. In particular, we sought to avoid genes that may be important for bacterial viability, such as metabolic and DNA replication genes as well as genes that encode components of major protein secretion systems, such as the Dot/Icm T4SS. The identified genomic regions were deleted using the suicide vector pSR47s which carries *sacB* conferring sucrose sensitivity, thereby providing selection pressure for double homologous recombination events. To enable homologous recombination, sequences flanking the genetic region to be deleted were cloned into the multi-cloning site of pSR47s. A detailed description of the methods used to generate the mutants is provided in [47]. Overall, we generated 13 mutants denoted ∆A–S, as well as a multi-region mutant that covered regions F, G, H, and I (∆FGHI) (Table 1, S1 Table).

**Table 1:** List of *L. pneumophila* 130b genomic mutants used in this study.

Strain	Deleted genes	Size of deleted regions (kb)	Number of deleted effector genes	Number of deleted non-effector genes
∆A	*lpw_00071–lpw_00211*	18.017	3	12
∆B	*lpw_04831–lpw_04861*	4.764	3	1
∆C	*lpw_11451–lpw_11581*	14.387	6	8
∆D	*lpw_16861–lpw_16971*	27.241	6	6
∆E	*lpw_19951–lpw_20201*	33.112	10	16
∆F	*lpw_2310–lpw_23561*	57.568	14	33
∆G	*lpw_26241–lpw_26431*	21.878	8	12
∆H	*lpw_26521–lpw_26861*	32.458	9	26
∆I	*lpw_27241–lpw_27551*	45.088	12	21
∆O	*lpw_0031–lpw_00451*	13.019	5	10
∆P	*lpw_00931–lpw_00961*	2.922	3	1
∆R	*lpw_0107–lpw_01121*	8.190	3	3
∆S	*lpw_02961–lpw_02981*	5.542	3	0
∆FGHI	Combination of F, G, H, and I deleted regions	156.992	43	92

To identify off-target effects such as the disruption of genes unrelated to the deleted regions and to confirm that the deletion was successful, whole genome sequencing was performed for each deletion mutant strain. This confirmed the successful deletion of all designated regions. However, genome sequencing also revealed single-nucleotide polymorphisms (SNPs) in seven mutants (S2 Table). One base deletion in ΔG led to a premature stop codon in the gene encoding for the DNA-binding response regulator *letA* (*lpw_28981*). In addition, a SNP in the T4SS-B *lvh* component *lvh1D4* was identified in ∆H. As *letA* is known to control the expression of virulence genes in *L. pneumophila* [48] and the Lvh type IV secretion system may affect the virulence of this strain, ∆G and ∆H were included for intracellular replication assays but excluded from analyses of macrophage transcriptional responses.

To ensure that the effector cluster deletions and other identified SNPs did not lead to a significant reduction in bacterial viability, bacterial growth in axenic media was monitored for 40 h. None of ∆C, D, E, F, G, H, I, O, P, R, S or ∆*legk2* showed major differences in replication *in vitro* compared to the wild-type strain (Fig 1A). Two mutants, ΔA and ΔB, showed a slight delay in log phase and lag phase growth respectively, but ultimately reached the same optical density as the wild-type strain (Fig 1A). Some small differences in stationary phase were also observed for ∆D, F, G, S and ∆FGHI, however, overall, we were confident that the deletion of selected effector-rich gene clusters did not greatly affect the viability of the bacteria *in vitro*.

**Fig 1:**
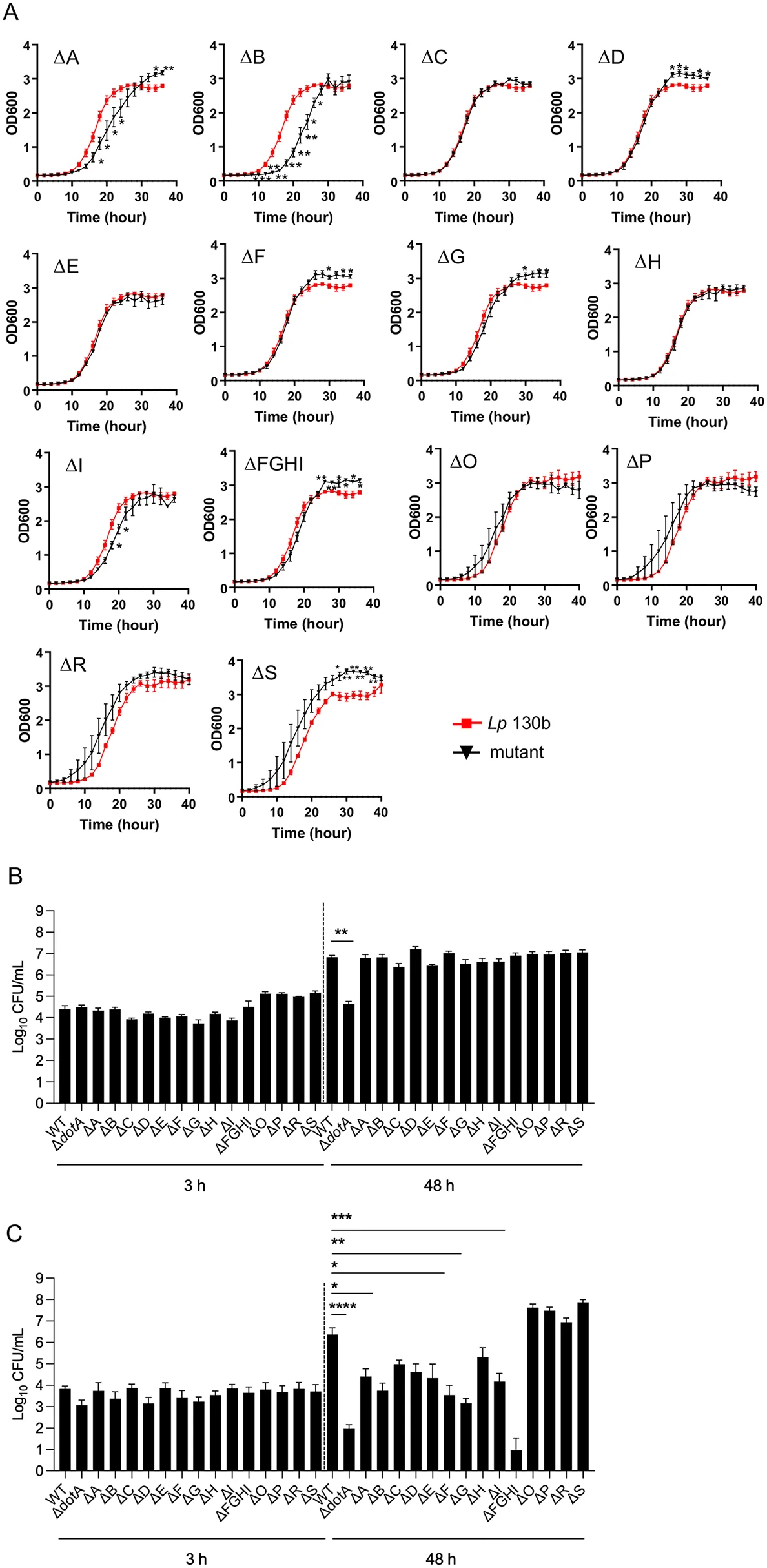
Creation and characterization of *L. pneumophila* genomic mutants. **A.** Viability of genomic deletion mutants in rich liquid medium (ACES broth). OD_600_ was measured every 2 h over a 40-hour time period (n = 3, mean ± standard error of the mean/SEM). *p < 0.05, **p < 0.01 (unpaired t-test). **B.** Replication of genomic deletion mutants in THP-1 macrophage, infected at an MOI of 10 (n > 3). Each bar represents the mean ± standard error of the mean (SEM). *p < 0.05, **p < 0.01 (Kruskal-Wallis with FDR correction). **C.** Replication of genomic deletion mutants in *Acanthamoeba castellanii*, infected at an MOI of 2 (n > 3). Each bar represents the mean ± standard error of the mean (SEM). *p < 0.05, **p < 0.01, *** p < 0.001, **** p < 0.0001 (Kruskal-Wallis with FDR correction).

The ability of the multi-deletion mutants to replicate intracellularly in THP-1 macrophages was then assessed using gentamicin protection assays. Bacterial colony forming units (CFU) were similar between wild-type and mutant strains after phagocytosis (3 h post-infection), confirming that all mutants were taken up by macrophages efficiently (Fig 1B). The Δ*dotA* mutant, which lacks a functional Dot/Icm T4SS and consequently the ability to replicate in both *A. castellanii* and THP-1, was used as a negative control. After allowing for two rounds of intracellular replication (48 h post-infection), all multi-deletion mutants were able to replicate competently in THP-1 macrophages. This included ΔFGHI, which despite the deletion of 134 genes, did not show a defect in intracellular replication compared to wild-type *L. pneumophila* after 48 h. This supports a previous multi-effector deletion study where a *L. pneumophila* mutant strain lacking 31% of the genome was still able to replicate efficiently in mammalian macrophages [43]. In summary, all the mutants were competent for replication in THP-1 cells, which further demonstrated the redundancy of the *L. pneumophila* genome.

In contrast to the macrophage replication phenotype, five multi-deletion mutants displayed a significant reduction in bacterial replication in *A. castellanii* (Fig 1C). At 48 h post infection, mutants ΔB, ΔF, ΔG, and ΔFGHI showed significantly reduced replication in *A. castellanii* compared to wild type 130b, while ΔA, ΔC, ΔD, ΔE, ΔH, ΔI, ΔO, ΔP, ΔR and ΔS could all replicate efficiently in *A. castellanii* (Fig 1C). The reduced replication of ΔB may be related to the extended lag phase observed during *in vitro* growth, although this did not affect replication of the mutant in THP-1 cells (Fig 1B). The phenotype of ΔG may be due to the premature stop codon introduced in *letA* which is known to influence *L. pneumophila* replication in amoebae [49]. The intracellular replication defect in *A. castellanii* varied for each mutant, ranging from ~ 10^3^–10^5^ lower in recovered bacterial CFU compared to wild type. ΔFGHI, which lacks genomic regions F, G, H and I had the most severe replication defect, comparable to Δ*dotA* mutant levels. This suggested that like Δ*dotA*, ΔFGHI may be unable to escape the endocytic-lysosomal pathway. In *A. castellanii,* CFUs at 3 h post infection displayed no difference in infection rate between mutant and wild type strains, indicating that the decreased CFU was not caused by reduced bacterial uptake (Fig 1C). This is consistent with the previous multi-effector deletion study [43] and other studies that report the attenuation of *L. pneumophila* mutants lacking Dot/Icm effectors in amoebae, but not in macrophages [39,43,50]. Overall, these results suggested that genes deleted in a subset of the mutants were critical for bacterial replication in *A. castellanii*

### The predicted amino acid transporter LPW_04851/Lpg0404 is required for replication in amoebae

We further investigated the basis for the replication defect of ∆B, which is missing only four genes (*lpw_04831*–*lpw_04861*) in *A. castellanii* (Fig 2A, Table 1). Other multi-deletion mutants lacking a similar number of genes did not show any similar attenuation in *A. castellanii* (∆O, ∆P, ∆R, ∆S, Fig 1C, Table 1). Single gene mutants of ∆B were generated, and we found growth of the single mutants *in vitro* was comparable to wild type 130b (Fig 2B). Intracellular replication assays for all single mutants were performed in *A. castellanii* and ∆*lpw_04851* showed significantly decreased CFU compared to wild type at 48 h post infection, while ∆*lpw_04831*, ∆*lpw_04841*and ∆*lpw_04861* replicated to wild type levels*,* indicating that *lpw_04851* was the factor responsible for the replication phenotype of ∆B (Fig 2C). To confirm the role of *lpw_04851,* plasmid pMMB207c-4xHA, also known as pHA or pICC562 [51], carrying the promoter region and the full coding sequence of *lpw_04851* was reintroduced into ∆*lpw_04851*. As a result, replication of ∆*lpw_04851* in *A. castellanii* was restored (Fig 2C).

**Fig 2:**
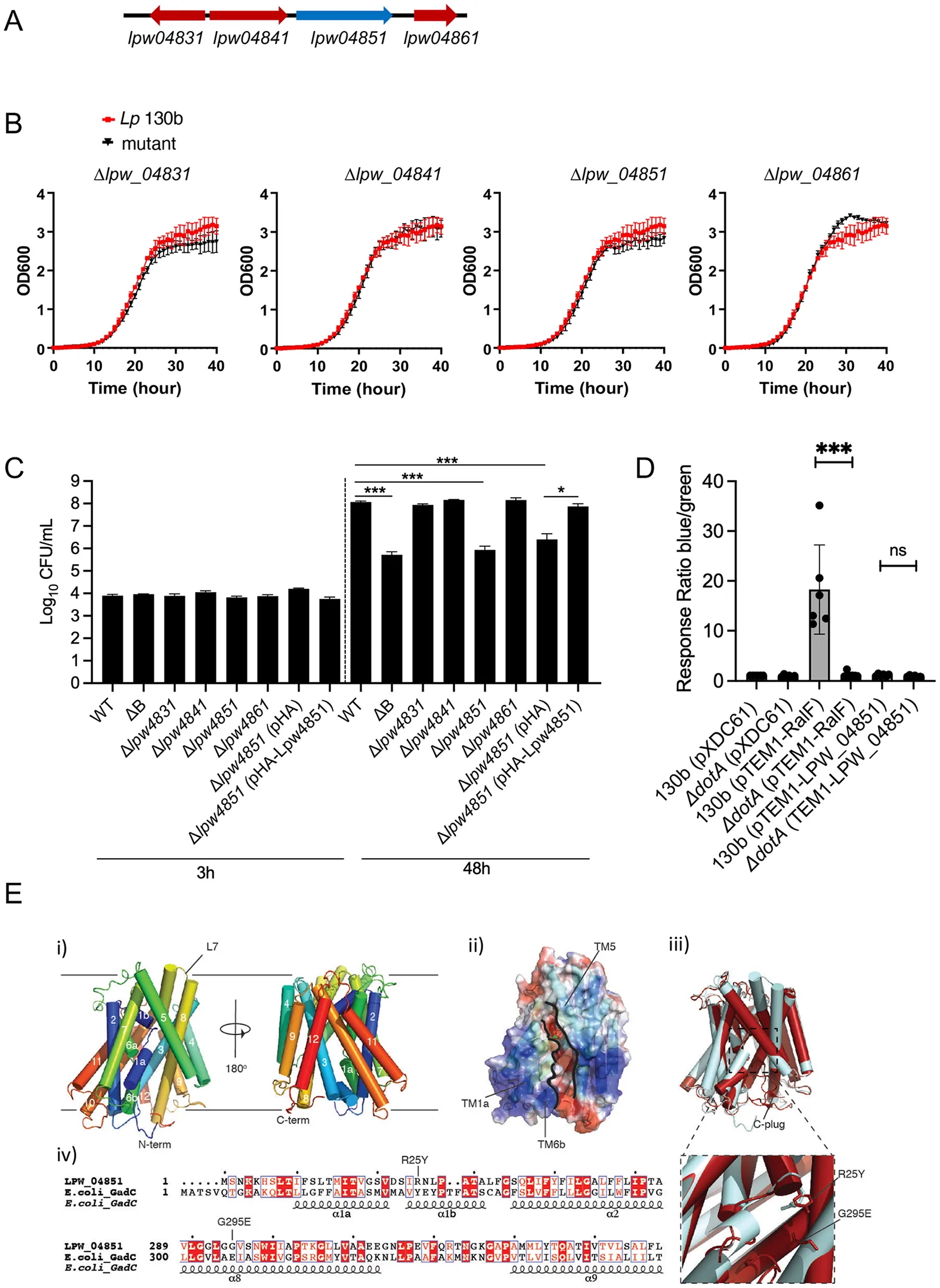
Replication of a *L. pneumophila lpw_04851* mutant in *A. castellanii.* **A.** Diagram of genomic region **B.** Red arrows represent the Dot/Icm effector-encoding ORFs, whereas the blue arrows represents the non-Dot/Icm effector-encoding ORFs. **B.** Viability of single deletion mutants of region B in rich liquid medium (ACES broth). OD_600_ was measured every 2 h over a 40-hour time period (n = 3, mean ± standard error of the mean/SEM). **C.** Replication of single deletion mutants of region B and the complemented *lpw_04851* mutant in *A. castellanii* infected at an MOI of 2 (n = 3, mean ± standard error of the mean/SEM). Statistical significance was determined using one-way ANOVA. **D.** TEM1 assay to test for translocation of LPW_04851. THP-1 cells were inoculated with derivatives of *L. pneumophila* as indicated at an MOI of 40 for 1 h. Data is expressed as the emission ratio of 450 nm to 520 nm. *P*-value from unpaired two tailed t-test, n = 6. ***indicates *p* value = 0.0009, ns, not significant **E.** Structural homology model of LPW_04851 **i)** Cartoon representation showing the 12 transmembrane (TM) helices of LPW_04851 **ii)** Electrostatic surface of LPW_04851 showing the negatively charged substrate binding path. **iii)** Superposition of LPW_04851 and *E. coli* GadC showing the absence of C-plug in LPW_04851 and the amino acid substitutions on the gating residues between the two transporters. **iv)** Sequence alignment of two regions between LPW_04851 and GadC showing the change in gating residues (R25Y and G295E; *Legionella* numbering).

According to homology detection by HMM-HMM comparison [52], the secondary structure of LPW_04851 is similar to the *E. coli* glutamate-GABA transporter GadC (E = 1.9 × 10^–43^). To determine if LPW_04851 was translocated into THP-1 cells, we generated a TEM1- LPW_04851 fusion protein and performed the CCF2-AM FRET based translocation assay as described previously [53]. However, we were unable to detect LPW_04851 translocation under these standard infection conditions (Fig 2D). We next constructed a homology model of LPW_04851 to gain insight into the structure and function of this protein. The model showed a canonical fold of the amino-acid-polyamine-organocation superfamily of membrane transporter, which possessed 12 transmembrane helices in an inward-open conformation similar to the crystal structure of *E. coli* GadC (Fig 2E) [54]. The substrate binding path of the channel is lined by a negatively charged surface contributed by the TM1a and TM6b helices (Fig 2E). Unlike the GadC structure, LPW_04851 lacks the C-terminal plug which occludes the opening on the cytoplasmic side of GadC. Moreover, amino acid substitutions were observed in several gating residues (R25Y and G295E; *Legionella* numbering) between LPW_04851 and GadC (Fig 2E). G295 of LPW_04851 is part of the TM8 gating helix containing significantly more glycine residues than GadC (Fig 2E). The absence of side chain in G295 in TM8 and the absence of the C-plug suggest the putative substrate may pass through more readily at this position of the channel. We concluded that LPW_04851 may contribute to nutrient acquisition by the bacteria during intracellular replication in *A. castellanii*, but that this was not required in THP-1 cells.

### Macrophages produce distinct transcriptomic responses during infection with effector cluster mutants

Many *L. pneumophila* effectors influence host processes but are dispensable for bacterial replication in mammalian macrophages [42,55,56]. Given that all the effector cluster mutants replicated to similar levels in THP-1 macrophages, we determined the macrophage gene expression responses upon infection with the mutants to understand if deletion of the effectors influenced host molecular signalling. Here we performed multiplex RNAseq analysis from mRNA isolated from THP-1 macrophages 18 h after infection with wild-type or mutant *L. pneumophila* strains to characterize the macrophage transcriptomic response. A multi-dimensional scaling (MDS) plot grouped uninfected THP-1 macrophages with macrophages infected with the *L. pneumophila* strain lacking a functional Dot/Icm T4SS (*ΔdotA*), and these were clearly separated from cells infected by the wild-type strain (Fig 3A). There was a distinct trend of segregation for each mutant infection. THP-1 macrophages infected with ∆I, ∆O, ∆P, and ∆R clustered close to the wild-type infected cells, whereas infection with ∆A, ∆B, ∆D, ∆S, and ∆FGHI clustered separately from the wild type-infected cells in the MDS plot. Cells infected with ∆C or ∆E created an additional separate cluster (Fig 3A). Among the genomic mutants, ∆FGHI lacked the highest number of effectors, and consistently, ∆FGHI-infected macrophages were segregated the furthest from the wild-type infected cells.

**Fig 3:**
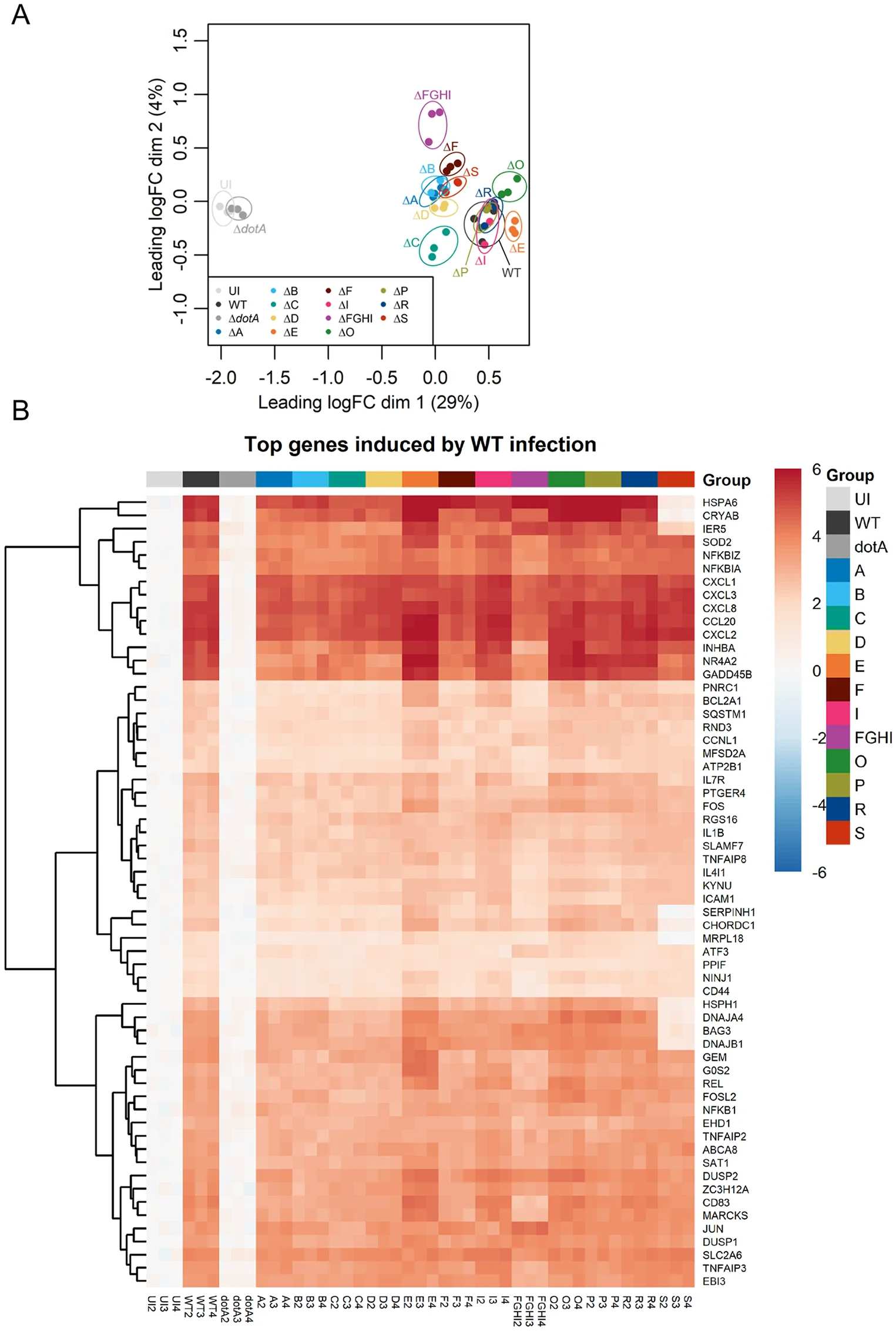
Transcriptomic response of THP-1 macrophages to *L. pneumophila* infection. **A.** Multidimensional (MDS) plot visualizes the difference in transcriptomic response of THP1 macrophages following infection by wild-type or *L. pneumophila* mutants at an MOI of 10 across two leading axes based on the top 500 most variable genes across all sample pairings*.* Different mutants are indicated in different colors as indicated. **B.** Heat map of the top 60 most differentially expressed genes during wild-type *L. pneumophila* infection compared to uninfected control. Top genes were selected based on the lowest FDR-adjusted p value. Scale was truncated to ± 6.

Overall, 755 genes were upregulated, and 106 genes were downregulated during infection with wild-type *L. pneumophila* compared to the uninfected control (S3 Table)*.* Compared to the uninfected control, macrophages infected by the avirulent ∆*dotA* mutant displayed no significant differentially expressed genes (S3 Table). Unsurprisingly, infection with wild-type *L. pneumophila* led to increased expression of pro-inflammatory genes, including the NF-κB regulators IκBα (*NFKBIA*), the NF-κB p105 and p65 proteins (*NFKB1, REL*), chemokines and cytokines (*CXCL1, CXCL3, CXCL8, IL1B*) (Fig 3B, S3 Table) and interferon-stimulated genes (ISGs, *IFIT1, IFIT3, STAT1*) (S3 Table). To better understand the relevance of the differentially expressed genes, a pre-ranked CAMERA gene set analysis was performed on the differential expression analysis results ranked by statistical significance, using the Hallmark and Reactome collections from MSigDB [57–60]. Gene set analysis revealed that infection with wild-type *L. pneumophila* resulted in the upregulation of the sets related to inflammatory response, NF-κB, and interferon responses (S1 Fig). In addition, we observed an upregulation of genes involved in the cellular response to heat gene set (S1B Fig), such as the heat shock protein family A (HSP70) member 6 *HSPA6,* the Hsp40 *DNAJA4*, and the co-chaperone *BAG3* (Fig 3B, S3 Table). The unfolded protein response (UPR) gene set was also upregulated by wild-type *L. pneumophila* infection (S1A Fig) as shown by the induction of the genes involved in UPR pathway, including PERK (*EIF2AK3*), CHOP (*DDIT3*), and ATF4 (*ATF4*) (S3 Table). *L. pneumophila* infection is known to induce various cell stresses, including the unfolded protein response (UPR) and the heat shock response (HSR) [61–63].

The transcriptional response of THP-1 cells to the genomic deletion mutants displayed some notable differences to wild type *L. pneumophila* in many of the “top 50” genes. This included ∆E, which exhibited significant up-regulation, ∆FGHI which showed significant downregulation and ∆S which displayed down regulation of a specific subset of genes (Fig 3B). Globally, compared to wild-type *L. pneumophila,* ∆A, ∆B, ∆D, ∆F, ∆FGHI, and ∆S all displayed downregulation of genes (Fig 4). For example, wild-type *L. pneumophila* robustly induced the expression of ISGs, whereas ∆A, ∆B, ∆D, ∆F, ∆FGHI, and ∆S elicited a comparatively weaker response (Fig 5A, S3 Table, S4 Table). Consistently, when compared to wild-type infected cells, infection by the mutants led to a reduction of the Hallmark interferon alpha and Reactome interferon alpha-beta gene sets (Fig 5A and 5B, S3 Table, S4 Table). Furthermore, THP-1 macrophages infected with ∆A, ∆B, ∆F, and ∆FGHI failed to upregulate the Hallmark interferon alpha gene set and Reactome interferon alpha beta signaling gene set when compared to the uninfected samples (S1A Fig, S1B Fig). While ∆D and ∆S induced higher expression of these gene sets compared to uninfected cells, the level of induction was significantly lower than wild-type infection (Fig 5B).

**Fig 4:**
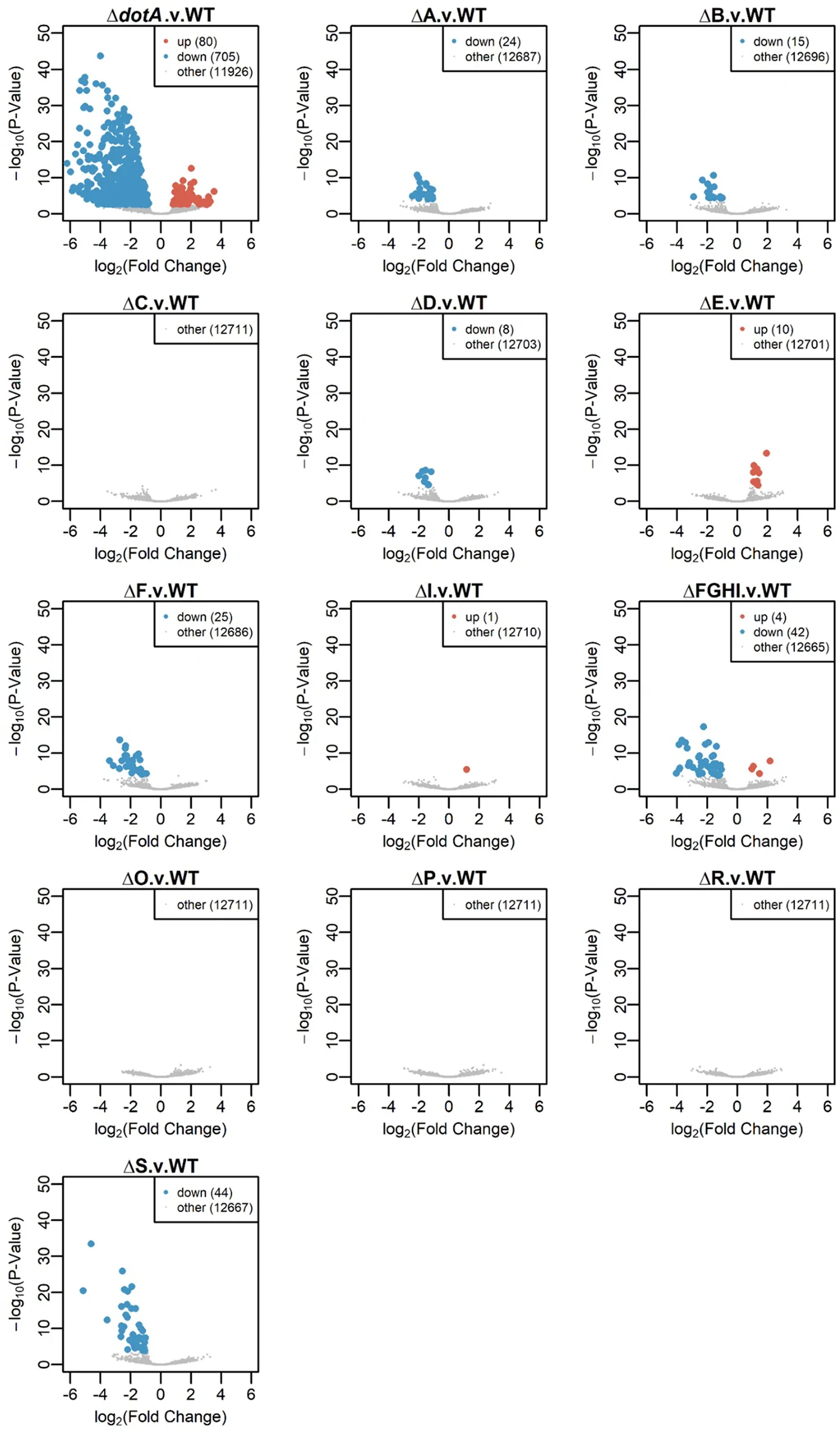
Comparison of differentially expressed genes in THP-1 cells during infection with genomic deletion mutants to wild-type *L. pneumophila* infection. Volcano plot showing the differentially expressed genes during infection by each mutant strain compared to wild-type *L. pneumophila* infected controls (treat fold-change = 1.5, FDR-adjusted p-value < 0.05). Red dots indicate genes significantly upregulated during mutant infection whereas blue dots indicate significantly downregulated genes during mutant infection. Macrophages were infected at an MOI of 10.

**Fig 5:**
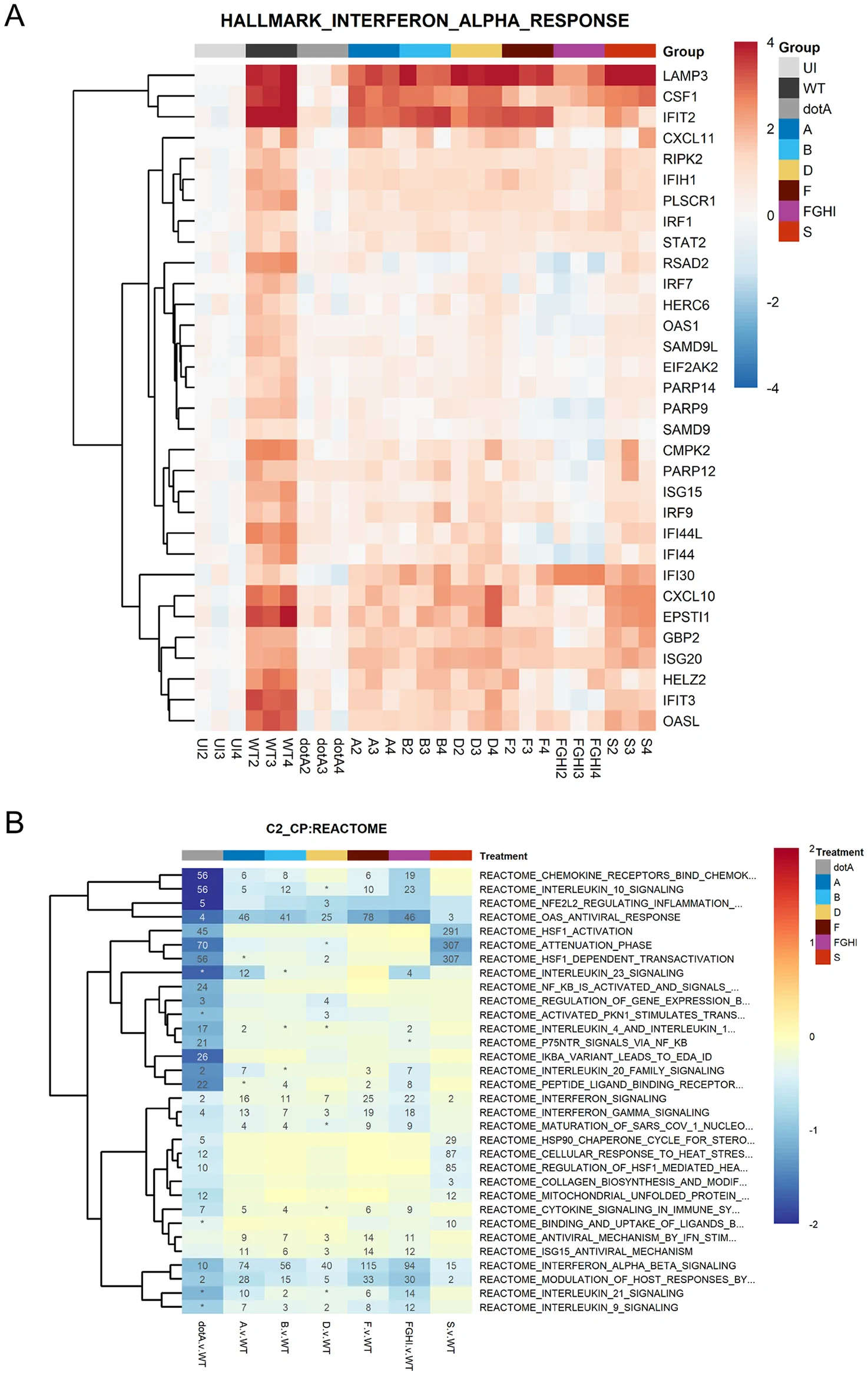
ISG response of THP-1 cells to infection with mutants ∆A, ∆B, ∆D, F∆, ∆FGHI, and ∆S. **A.** Heat map of the interferon-stimulated genes (ISGs) differentially expressed during wild-type *L. pneumophila* infection (treat fold-change = 1.5). The color gradient shows the log2 count per million (log2 CPM) of genes in cells infected by different *L. pneumophila* strains as indicated in the legend at an MOI of 10. Scale was truncated to ± 2. **B.** Heat map of Reactome gene sets that were significantly changed during infection with different *Legionella* mutant strains compared to wild-type *L. pneumophila* 130b infection. Color gradient indicates the average log2 fold change of the gene set, whereas numbers indicate the –log10(p-value). Scale was truncated to ± 2.

Strikingly, compared to wild type *L. pneumophila* and the other mutants, ∆S failed to upregulate the expression of genes encoding known heat shock proteins and chaperones, including *HSPA6, CRYAB, IER5, SERPINH1, CHORDC1, HSPH1, DNAJB1, BAG3* and *DNAJA4* (Fig 3B, Fig 6). CAMERA gene set analysis further revealed that ∆S infection led to a marked downregulation of the terms related to the activity of HSF1, as well as the cellular response to heat stress when compared to wild-type infection (Fig 6). Consistent with this observation, the bioinformatics tool HSF1Base [64] revealed that 34 out of the 44 genes downregulated in ∆S infection are regulated by HSF1 (S3 Table). Also, in contrast to the other mutants, ΔE-infected cells showed a modest upregulation of ten genes, but no downregulation of genes compared to wild type *L. pneumophila* (Fig 4). Reactome gene set analysis suggested upregulation of several HSF1-related terms, perhaps due to the robust upregulation of the HSP20 small heat shock protein-encoding gene *HSPB1* and the chaperone alpha-crystallin B *CRYAB* (S2A Fig, S4 Table). Although ∆O and ∆P caused a modest increase in *HSPB1* and *CRYAB* expression (S2B Fig), due to the low log2 fold change, this increase was deemed insignificant in the RNAseq analysis.

**Fig 6:**
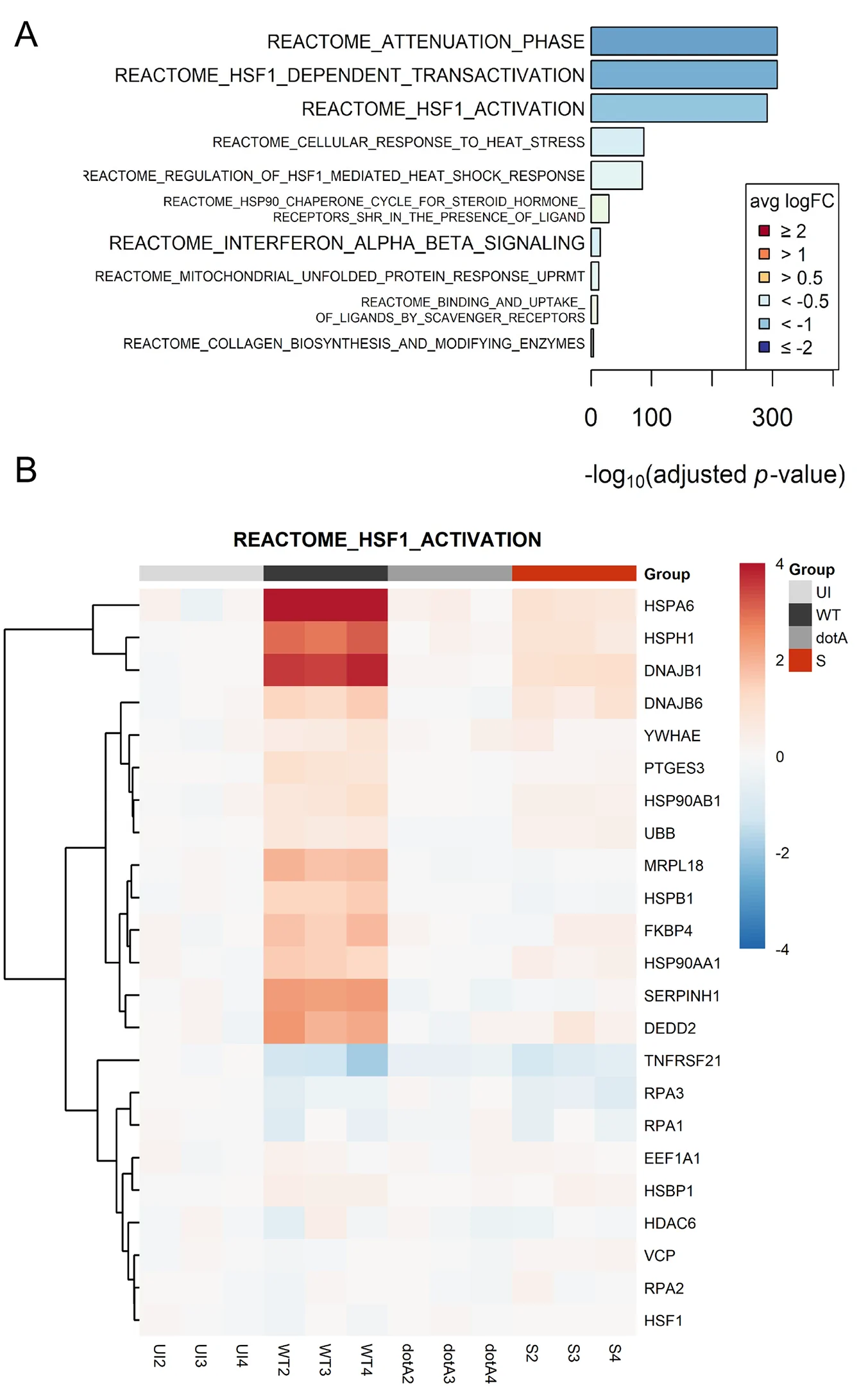
Infection with ∆S induced an attenuated heat shock response compared to infection with wild-type *L. pneumophila.* **A**. The top ten Reactome gene sets downregulated by ∆S infection based on FDR-adjusted P-value. Only terms with FDR-adjusted P-value < 0.05 are shown. Bars represent –log10 of FDR-adjusted p-value. **B.** Heat map of genes in the Reactome gene set HSF1_ACTIVATION. Colour gradient represents the log2 count per million (log2 CPM) of genes in cells infected by different *L. pneumophila* strains as indicated in the legend at an MOI of 10. Only genes determined to be significantly reduced in ∆S vs wild-type comparison (treat fold-change = 1.5) are shown. Scale was truncated to ±2.

THP-1 cells infected with mutants ∆I, ∆O, ∆P, and ∆R showed no differentially expressed genes consistent with the fact that these mutants clustered with wild type infected cells (Fig 3A, Fig 4). Despite clustering slightly away from the wild-type samples, ∆C showed only modest differences in differentially expressed genes that were not significant in our analysis (Fig 3A, Fig 4). Overall, this analysis showed that the deletion of effector genes can influence the macrophage transcriptional response despite having no obvious impact on intracellular replication and bacterial load in THP-1 cells.

**Role of the HSF1-induced heat shock response during *L. pneumophila* infection**.

Of all the genomic mutants, ∆S displayed the most distinct transcriptomic response compared to wild-type infection. Therefore, we decided to determine which Dot/Icm effector(s) in ∆S triggered the host heat shock response during infection. To this end, individual effector genes in region S were identified and deleted (Fig 7A). After 18 h of infection with the single gene deletion mutants, we observed that a mutant lacking *legK4,* but not other mutants, was unable to upregulate the host heat shock response (Fig 7B). LegK4 is an effector kinase known to phosphorylate and inactivate host HSP70 [24]. Here complementation of the mutant with a plasmid encoding HA-tagged *legK4* (pHA-LegK4), but not a kinase-dead mutant (pHA-LegK4_D271A_), restored the ability of *L. pneumophila* to trigger the host heat shock transcriptional response (Fig 7C). This was not due to differences in expression of HA-LegK4 and HA-LegK4_D271A_ from the expression plasmids (Fig 7D) and could indicate that the inactivation of HSP70 by LegK4 drives a proteotoxic stress response that results in a broader host heat shock response.

**Fig 7:**
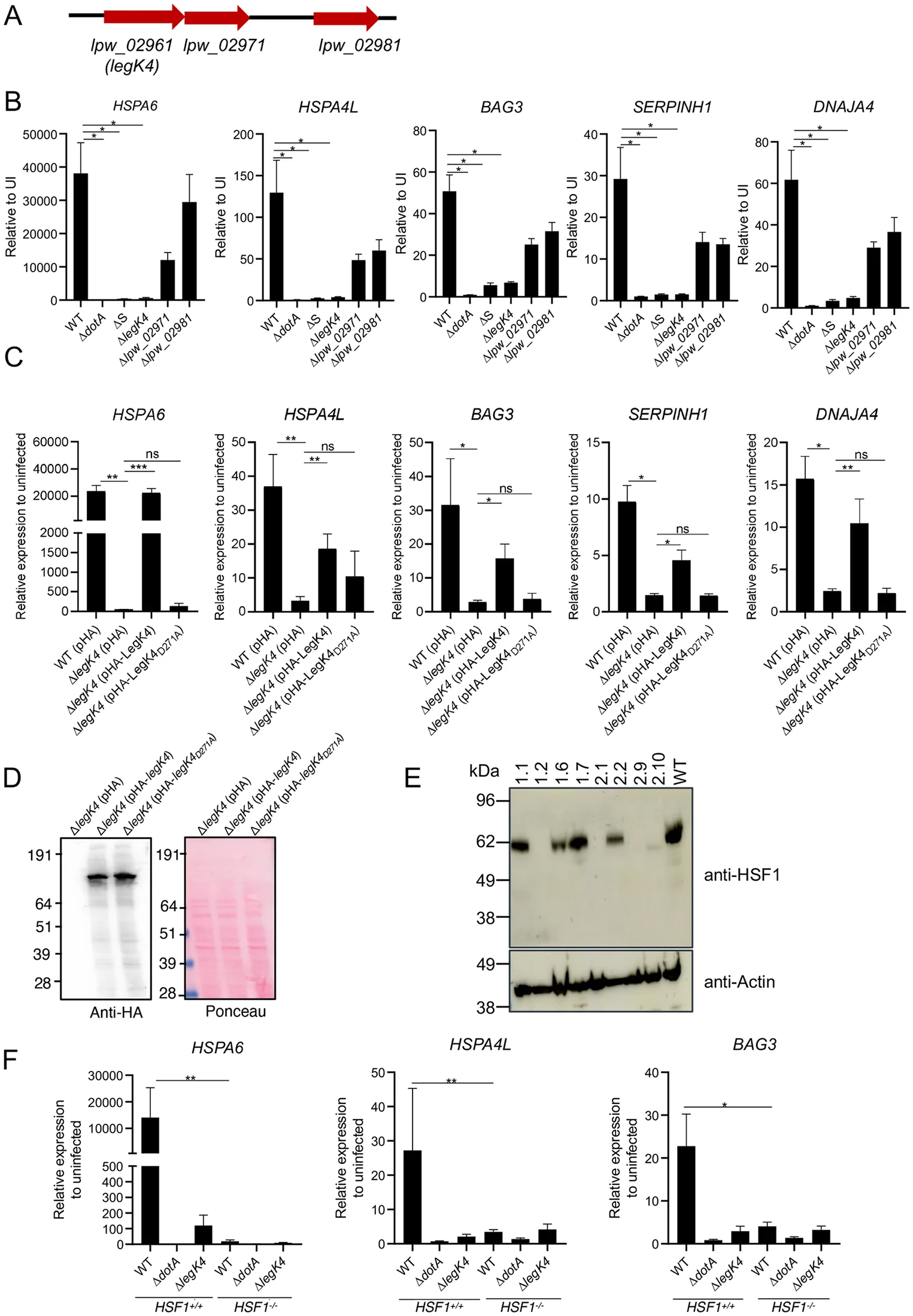
LegK4 is responsible for the induction of host heat shock response through host HSF1 during *L. pneumophila* infection. **A.** Diagram of genes that are deleted in genomic region **S.** Red arrows represent Dot/Icm effector ORF. **B.** qRT-PCR of a panel of top heat shock proteins and chaperones that were downregulated by ∆S after 18h of infection by single gene mutants from genomic region S at an MOI of 10. Bars indicate the mean of expression relative to uninfected control ± SEM (n > 4). Statistical significance was determined with Mann-Whitney U-tests (* p < 0.05). **C.** qRT-PCR of a panel of top heat shock proteins and chaperones that are downregulated by ∆S after 18h of infection by the complemented ∆*legK4* strains at an MOI of 10. Bars indicate the mean of expression relative to uninfected control ± SEM (n > 4). Statistical significance was determined with Mann-Whitney U-test (* p < 0.05, ** p < 0.01, *** p < 0.001). **D.** Expression of HA-LegK4 in *L. pneumophila* culture. Δ*legK4 L. pneumophila* 130b carrying either p4HA, p4HA-LegK4, or p4HA-LegK4_D271A_ were cultured for 18 h in ACES at 37˚C with agitation. Expression of HA-LegK4 was then induced by adding 1 mM IPTG for 3 **h.** Immunoblot was performed using anti-HA antibody (left panel) and Ponceau stain as loading control (right panel). **E.** Expression of HSF1 in the HSF1 CRISPR KO cell lines. Two independent guide RNA (gRNA) pairs was used to generate CRISPR KO clonal cell lines, and at least one clone from each gRNA pair was used. Numbers indicate the clone number, WT refers to lysate of unmodified THP1. The first number in the clone number refers to the gRNA number, whereas the second number refers to the colony number. Clones 1.1 and 2.2 were used as *HSF1*^*+/+*^ control, whereas clones 1.2, 2.1, and 2.9 were used as the *HSF*^*-/-*^ samples. **E.** qRT-PCR of a panel of top heat shock proteins and chaperones in HSF1 CRISPR KO cell lines. Bars indicate the mean of expression relative to uninfected control + /- SEM. Data from all clones of each genotype was pooled for analysis (n = 2 *HSF1*^*+/+*^ clones and n = 3 *HSF1*^*-/-*^ clones from 2 cohorts). Statistical significance was determined with Mann-Whitney U-test (* p < 0.05, ** p < 0.01).

The heat shock response in mammalian cells is regulated by the major transcription factor Heat Shock Factor 1 (HSF1) [65]. To examine if the upregulation of heat shock by *L. pneumophila* occurred through this canonical pathway, we generated a knock-out of *HSF1* in THP-1 cells using CRISPR (Fig 7E). In the absence of *HSF1*, wild type *L. pneumophila* was unable to upregulate the expression of heat shock genes in infected THP-1 cells as occurred in wild type macrophages (Fig 7F), indicating that the broader impact of LegK4 on heat shock depends on the canonical transcription factor HSF1. To determine the effect of heat shock activation *L. pneumophila* intracellular replication, we examined replication of the ∆*legK4* single mutant strain in THP-1 cells and *Acanthamoeba castellanii*. ∆*legK4* did not show any difference in replication compared to wild type *L. pneumophila* in either host cell type (Fig 8A and 8B). In addition, infection with Δ*legK4* caused a similar level of cytotoxicity in THP-1 macrophages compared to wild type *L. pneumophila* (S2C Fig). This is not surprising, as *L. pneumophila* Dot/Icm effectors are known to have redundant effects and no replication defect was reported previously for *legK4* mutants in amoebae [66]. Therefore, to investigate the role of the heat shock response in *L. pneumophila* infection, we examined intracellular replication of *L. pneumophila* in *HSF1* deficient THP1 macrophages. Uptake of *L. pneumophila* was reduced at 3 h and bacterial load was reduced at 24 h in *HSF1* deficient cells compared to wild type THP-1 macrophages. This effect was modest but reproducible. However, by 48 h post-infection comparable bacterial numbers of both wild type *L. pneumophila* and *legK4* mutant were observed in *HSF1* deficient cells and wild type THP-1 cells (Fig 8C). We did not observe any significant difference in cytotoxicity of infected cells between wild type and *HSF1* deficient macrophages (S2D Fig). These results suggested that the heat shock response may aid initial uptake of *L. pneumophila* and that this may influence intracellular bacterial numbers in the early stages of infection.

**Fig 8:**
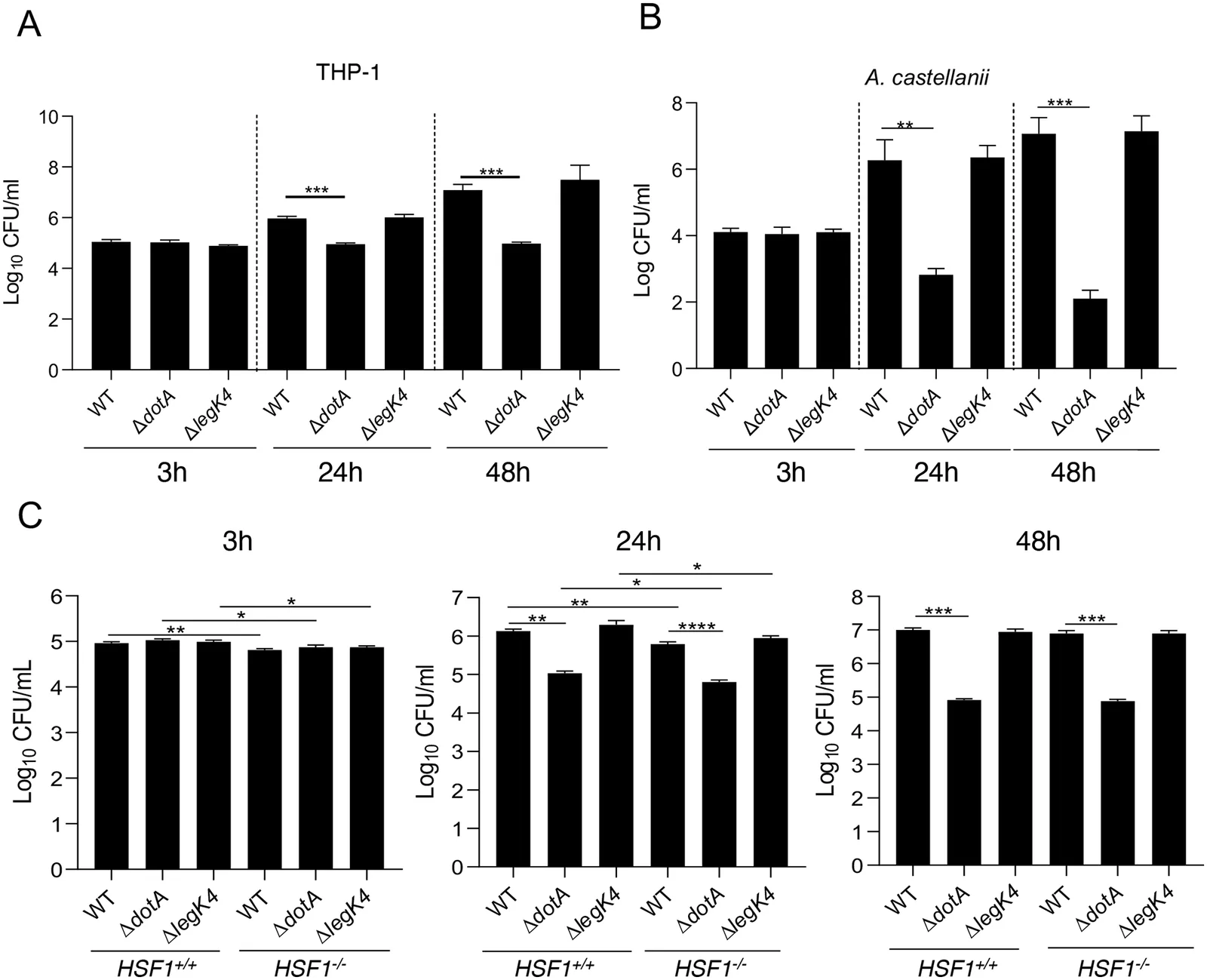
LegK4 is dispensable for *Legionella* replication in THP-1 and *A. castellanii.* **A.** Replication of wild-type *L. pneumophila* 130b and mutant strains in THP-1 macrophages infected at an MOI of 5 (n ≥ 3) over 48h. Bars represent the means and error bars represent standard error of the mean. Statistical significance was determined using unpaired t-tests (* p < 0.05, ** p < 0.01, *** p < 0.001). **B**. Replication of wild-type *L. pneumophila* 130b and mutant strains in *A. castellanii* infected at an MOI of 2 (n = 3) over 48h. Bars represent the means and error bars represent standard error of the mean. Statistical significance was determined using unpaired t-tests (* p < 0.05, ** p < 0.01, *** p < 0.001). **C.** Replication of wild type *L. pneumophila* and mutants in *HSF1 + /+* or *HSF1-/-* THP-1 cells infected at an MOI of 5 over 48h. Two independent guide RNAs (gRNAs) were used to generate the CRISPR KO cell lines, and at least one clone from each gRNA was used. Data from all clones of each genotype was pooled for analysis (n = 2 *HSF1*^*+/+*^ clones and n = 3 *HSF1*^*-/-*^ clones from 4 cohorts). Statistical significance was determined using Mann-Whitney U-tests (* p < 0.05, ** p < 0.01, *** p < 0.001).

Despite HSF1 driving strong transcriptional response to *L. pneumophila* infection, at 8 h and 18 h after infection, we did not see a difference in the protein levels of the heat shock factors Hsc70 and Hsp90 in THP-1 cells infected with wild-type or the *legK4* mutant, suggesting that the transcriptional activation observed in response to LegK4 does not lead to lasting proteomic change (S3A Fig). This could suggest that *L. pneumophila* counteracts HSF1 activation of the heat shock response by blocking protein synthesis using effectors that act redundantly with LegK4 (S3B Fig) [23–25].

## Discussion

Dot/Icm effectors are key virulence determinants during *L. pneumophila* infection. However, the functions of the majority of effectors are yet to be fully characterized. This is due largely to functional redundancy of the extensive repertoire of 330 bioinformatically- and experimentally identified effectors and the permissive nature of many laboratory adapted macrophage cell lines, such as THP-1 cells, with the result that the deletion of one Dot/Icm effector gene rarely results in a defect in bacterial replication [67]. Adding to the complexity, Dot/Icm effectors can regulate each other (termed metaeffectors), for example LubX degrades SidH using the host proteasomal degradation system [68].

To explore the collective impact of Dot/Icm effectors on the macrophage transcriptional response, in this study we created a collection of 14 multi-effector deletion mutants in *L. pneumophila* strain 130b and compared the effect of different mutants on macrophage signalling pathways. Although effector rich regions were prioritized for deletion, some non-effector genes were also unavoidably targeted through this process. We confirmed that all mutants were replication competent for *in vitro* growth, although modest differences in stationary phase were observed for ∆D, F, G, S and ∆FGHI, perhaps indicating differences in managing nutrient depletion, and/or reduced levels of inhibitory metabolites compared to wild type *L. pneumophila*. However, this was not examined further here. We also observed that ΔA exhibited slower exponential phase growth and ΔB exhibited an extended lag phase compared to wild type 130b. The gene *lpw_00091*, encoding the RNA-binding chaperone Hfq is missing in ∆A and mutants lacking *hfq* exhibit a delay in the exponential phase growth [69], similar to our observation. However, *hfq* mutants remain competent in the replication in both macrophage and amoebae at 37°C [69]. On the other hand, ΔB showed an extended lag phase in the nutrient-rich media. While the lag phase is still poorly studied, it is predicted to allow the bacteria to adapt to new culture environments [70,71]. ∆B also displayed a significant reduction in replication in *A. castellanii* but not THP-1 cells. We identified that a putative glutamate transporter, encoded by *lpw_04851,* was responsible for this phenotype. During intracellular replication, *L. pneumophila* utilizes host-derived amino acid transporters for nutrient acquisition in the LCV. *L. pneumophila* up-regulates transcription of various host transporters during infection including SLC1A5 and SLC7A5, enabling *L. pneumophila* to import amino acids from the cytoplasm into the LCV lumen [72,73]. ^13^C isotopologue profiling has shown that *L. pneumophila* acquires Ala, Asp, Glu, Ser, Phe, Tyr, Pro, and Gly from *A. castellanii* and incorporates these amino acids directly into bacterial proteins, even though the bacteria harbor biosynthetic pathways for Ala, Asp, and Glu [74]. Glutamate is a crucial amino acid for the lifecycle of *L. pneumophila in vitro* [75,76]. However, glutamate is dispensable for bacterial replication in Mono Mac 6 human monocytes, suggesting a metabolic transition when replicating intracellularly [72]. Consistent with previous metabolic profiling of *L. pneumophila* in *A. castellanii* and human monocytes, our finding of the distinct replication phenotypes of ∆B in *A. castellanii* and THP-1 cells, suggests that replication in different hosts requires the adaptation of bacterial metabolism [74]. Further studies will determine the function of LPW_04851/Lpg0404 and hopefully provide deeper insight into host-specific bacterial metabolic routes.

Broadly, our study affirmed the functional redundancy within the Dot/Icm effector repertoire in mammalian cells, as the mutant that covered the greatest number of effectors, ΔFGHI, which lacks 43 effector genes, did not show any reduction in bacterial replication in THP-1 macrophages. This is consistent with a previous study of gene essentiality by O’Connor *et al* performed in the *L. pneumophila* Philadelphia background [43], which reported that the loss of up to 31% of the genome did not affect *L. pneumophila* replication in the mammalian macrophages. In contrast, ∆FGHI was highly attenuated in *A. castellanii.* Host-dependent differences have been reported previously, where Dot/Icm effector genes are required for replication in protozoa but dispensable for replication in mammalian cells [33,39,50]. Indeed, our results support findings from several other studies exploring the contribution of Dot/Icm effector proteins to intracellular replication in amoebae. For example, ∆F, which was attenuated for replication in *A. castellanii* includes *legk2* (*lpw_23101/lpg2137*) and two separate studies showed that *legK2* is required for replication in *A. castellanii* and *Dictyostelium* respectively [66] [77]. ∆F also lacks *sidJ* (*lpw_23291/lpg2155*) and the Sde family effectors which contribute to intracellular replication in amoebae [21,78,79]. Additionally, the effector MesI (*lpw_27311/lpg2505),* which is deleted in ∆I, was reported to be required for replication in *A. castellanii* [39]. Although we did not see similar attenuation here for ∆I, the deletion of *mesI* could be contributing to the dramatic attenuation of ∆FGHI in *A. castellanii*.

It should be noted here that we initially generated the multi-region mutant ∆FGHI to evaluate whether a greater phenotype would be observed compared to the smaller deletions. However, this also made interpretation more complex, due to interactions between the products of the genes deleted in each smaller region. Therefore, work is currently still underway to fully investigate the phenotype of ∆FGHI.

Several of our multi-gene deletions overlap with those described in O’Connor *et al* [43]. For example, our ∆C deletion is within the Cluster 2ab mutant in the O’Connor study, and neither the ∆C mutant nor the Cluster 2ab mutant showed attenuation for replication in macrophages or amoebae. Our ∆D deletion is within Cluster 3 and while ∆D was not attenuated for replication in macrophages or *A. castellanii*, the Cluster 3 mutant did show attenuation in *A. castellanii*, suggesting genes outside the ∆D island were required for replication in amoebae. Our ∆E deletion is within Cluster 4a, and while neither mutant was defective for replication in macrophages, the Cluster 4a mutant was attenuated in *A. castellanii*. Likewise, our ∆I deletion is within Cluster 7a, and the Cluster 7a mutant, which lacks *mesI,* was attenuated in *A. castellanii* whereas ∆I was not. One further anomaly was that Cluster 6a, which encompasses the ∆G deletion, did not show attenuation in any host cell type, whereas ∆G was attenuated in *A. castellanii.* However, as mentioned, we discovered that ∆G acquired a base deletion in *letA*, which probably explains the attenuation observed. These comparisons may help future studies pinpoint both effector and non-effector genes essential for *L. pneumophila* replication in amoebae as well as possible strain to strain variation.

Although the loss of large genomic regions in mutants ∆A, ∆B, ∆D, ∆F, or ∆FGHI did not result in a replication defect in macrophages, these mutants induced an attenuated ISG response. The simplest explanation for this phenotype is that perhaps the mutants have a subtle reduction in replication that was not detected through the gentamicin protections assays used here. However, since mutant ∆D displayed a modest increase in bacterial CFU, and none of the mutants showed reduced CFU after 48h of infection, we feel that this is unlikely to be the sole explanation. This phenotype might also stem from the host recognition of effector proteins, or the biochemical activity of the effector proteins, collectively resulting in an effector-triggered immune (ETI) response. While ETI was first described in plants, this phenomenon has previously been reported during *L. pneumophila* infection. For example, the Dot/Icm effector LamA was reported to promote glycolysis in amoebae to increase the availability of glucose [80]. Another notable example is the activation of host immune response through Dot/Icm effectors that inhibit host translation. In response to the translation block, MAPK signalling is activated independently of Toll-like receptor (TLR) and nucleotide-binding oligomerization domain-like (NOD) receptor signalling, as well as a slower replenishment of the nuclear factor-kappa B (NF-κB) inhibitor IκB, resulting in a sustained NF-κB response [81,82]. Together, this results in the superinduction of mRNA and the bypassing of the translational block. At this stage the reason behind the attenuated ISG transcriptional response across several genomic deletion mutants is unclear but warrants further investigation beyond this study.

In addition to the inflammatory response, *L. pneumophila* infection also strongly induced the heat shock response (HSR) [62,63]. HSR is a highly conserved protective response against various stresses, including high temperature, infection, and toxic substances. In mammalian cells, HSR is regulated by the transcription factor Heat Shock Factor 1 (HSF1), which upon activation elevates the expression of a diverse range of proteins, in particular the heat shock proteins (HSPs), such as HSP70, HSP90, and HSP40 [65]. Under physiological condition, HSF1 is held in an inactive state by HSP70 and HSP90. The accumulation of misfolded proteins prompts HSP70 and HSP90 to release HSF1 and bind to misfolded proteins. HSF1 then trimerizes and translocates to the nucleus to upregulate the expression of chaperones and co-chaperones. Our finding of strong host HSR induction corroborates a previous study that reported induction of the host HSR by the Dot/Icm effector SidI, which glycosylates the eukaryotic elongation factor 1A (eEF1A). SidI was reported to stall protein synthesis leading to ribotoxic stress and the upregulation of HSF1 expression [25,62]. However, the deletion of *sidI* by Shen *et al.* did not restore HSF1 activation back to uninfected levels [62]. In addition, the homolog of *sidI* in *L. pneumophila* 130b (*lpw_27301*) was missing from our ΔI and ΔFGHI mutants, yet these mutants still induced a HSR similar to wild type 130b. Instead, we found that the deletion of *legK4* completely eliminated induction of the host HSR during *L. pneumophila* infection. This could be complemented by reintroducing wild type *legK4*, but not the kinase-dead (D271A) mutant, thus highlighting the importance of the kinase activity of LegK4 for driving the host HSR. Although not directly shown in our study, triggering of the HSR may occur through LegK4-mediated phosphorylation and inactivation of host HSP70 [24]. HSP70 is responsible for the *de novo* folding of proteins, as well as correcting misfolded proteins [83]. If HSP70 is sufficiently impaired, misfolded proteins accumulate, leading to the activation of HSF1 and the HSR. However, despite inducing a strong transcriptional HSR, protein levels of HSC70 and HSP90 did not appear to be altered in wild type versus ∆*legK4* THP-1 infection over time. Both wild type and ∆*legK4* mediated translational suppression at 8 h and 18 h after infection, thereby preventing a functional HSR to *Legionella* infection. Hence, while LegK4 activates the HSR, *L. pneumophila* also blocks the ability of the macrophage to execute the HSR by preventing translational upregulation of heat shock proteins. In the early stages of infection, we found that the deletion of *HSF1* from THP-1 cells modestly but reproducibly reduced bacterial numbers upon uptake and in the early stages of replication, but CFU recovered by 48h post-infection. While our experiments did not distinguish between reduced uptake in *HSF1* deficient cells and possible subsequent effects on intracellular replication, future work could focus on dissecting the importance of early HSF1 activation before global translation inhibition is observed*.*

Overall, our results support the utility of multi-effector mutants to identify Dot/Icm dependent phenotypes [43]. Despite reaching similar levels of intracellular replication in THP-1 cells, several of the multi-effector deletion mutants induced an altered the macrophage transcriptional response compared to wild type infected cells, demonstrating that the presence of Dot/Icm effector proteins influences cell intrinsic and innate immune signalling. The multi-effector mutants are thus useful tools to dissect Dot/Icm effector phenotypes, effector triggered immunity and meta-effector activities.

## Materials and methods

### Bacterial strains and growth conditions

Bacterial strains and plasmids used in this study are respectively listed in S6 Table and S7 Table. *Escherichia coli* strains were grown in Luria-Bertani broth or agar (1% tryptone, 1% NaCl, 0.5% yeast extract, and 2% agar where appropriate). *L. pneumophila* were grown in either buffered charcoal yeast extract (BCYE) agar or N-(2-acetamido)-2-aminoethanesulfonic acid (ACES)-buffered yeast extract broth supplemented with 1 mM iron(III) nitrate (Fe(NO_3_)_3_) and 1 mM L-cysteine hydrochloride. *E. coli* and *L. pneumophila* cultures were incubated at 37°C for respectively 1 day and 3 days. Liquid cultures were incubated at 37°C for 18–20 h with shaking at 200 rpm.

### *L. pneumophila* mutant generation

Bioinformatics analysis to determine the genomic regions, cloning of suicide vector, and the mutagenesis of *L. pneumophila* were performed as previously published [47]. The flanking regions were amplified using the primers detailed in S8 Table and joined together using overlap PCR. The product was then digested with the appropriate restriction enzymes and ligated into pSR47s. The plasmid was then confirmed using Sanger sequencing using primers pSR47s F/R and an additional internal primer as detailed in S8 Table. One microgram of each plasmid was then transformed into *L. pneumophila* 130b and plated onto BCYE plus 100 μg/mL kanamycin. Colony PCR was performed to select for the colonies that had recombined the plasmid into the genome. Sucrose selection was then performed by plating onto BCYE + 5% sucrose to drive the second homologous recombination event. Colony PCR was again performed to select for the mutants.

### *L. pneumophila* complementation

The IPTG-inducible plasmid pMMB207c-4xHA (S7 Table) was used for the trans-complementation of *legK4*. The coding sequence (CDS) of *legK4* was amplified from the genomic DNA (gDNA) of *L. pneumophila* 130b using primer pairs LegK4 F/R (S8 Table) and ligated to pMMB207c-4xHA vector. Site-directed mutagenesis was performed using the Stratagene QuikChange II Site-Directed Mutagenesis kit according to the manufacturer’s protocol. Primer pairs D271A F/R were used to generate the mutant version of pMMB207c-4xHA:*legK4* (S8 Table). After confirmation using Sanger sequencing, 200 ng of each plasmid was transformed into *L. pneumophila* Δ*legK4* mutants by electroporation at 2.3 kΩ and plated on to BCYE plates containing chloramphenicol (6.25 μg/mL). Colonies were then screened using colony PCR using primers pMMB207c F and R.

### Whole genome sequencing

All mutant strains were confirmed using whole-genome sequencing. Draft genomes were sequenced using an Illumina NextSeq platform with Nextera XT libraries, as per manufacturer’s instructions. Single Nucleotide Polymorphisms (SNPs) within the draft genomes were detected by Snippy v3.2 [84] using the *L. pneumophila* 130b genome sequence (GenBank Assembly accession: GCA_000211115.2) as a reference. When required, Illumina reads were assembled using SPAdes v3.7.1 [85].

### Cell culture conditions

THP-1 monocytes (ATCC TIB-202) were maintained in Roswell Park Memorial Institute (RPMI) 1640 (Invitrogen) plus 10% foetal calf serum (FCS, Invitrogen). Prior to infection, the monocytes were differentiated into macrophages by adding 50 ng/mL of phorbol 12-myristate 13-acetate (PMA), seeded at 5x10^5^ cells/well in 24-well plates, and incubated at 37°C, 5% CO_2_ for 3 days. *Acanthamoeba castellanii* strain Neff (ATCC® 30010) were sustained in Peptone Yeast Glucose (PYG, 2% peptone, 0.1% sodium citrate, 0.1% yeast extract) medium supplemented with 0.1 M glucose, 0.4 mM CaCl_2_, 2.5 mM KH_2_PO_4_, 4 mM MgSO_4_, 2.5 mM Na_2_HPO_4_, 0.05 mM Fe_4_O_21_P_6_ at room temperature. Prior to *L. pneumophila* infection, *A. castellanii* were seeded at 2 × 10^5^ cells/well in 24-well plates. During infection, the cells were incubated in the minimal medium (PYG without glucose, yeast extract, and peptone) at either 37°C or 30°C as indicated in the text.

### *L. pneumophila* infection and replication assay

Differentiated THP-1 macrophages or *A. castellanii* were infected by stationary phase *L. pneumophila* and spun down at 1,100 rpm for 5 minutes to synchronize the infection. Phagocytosis was allowed to happen for 2 h at 37°C, 5% CO_2_ for THP-1 macrophages or as indicated in text for *A. castellanii*, followed by 1 h of gentamicin treatment (100 μg/mL) to kill extracellular bacteria. After washing off the gentamicin, the cells were incubated at 37°C, 5% CO_2_ until the timepoint indicated in text. For infection with strains carrying the IPTG-inducible plasmids, 1 mM IPTG was added to the infected cells after the gentamicin wash step to induce expression. For the replication assay in THP-1 macrophages, at each timepoint cells were lysed with 0.05% digitonin for 5 minutes at room temperature, harvested into sterile Eppendorf tubes, and vortexed. In *A. castellanii*, cells were harvested in sterile dH_2_O and vortexed to release the bacteria. Serial dilutions were performed, and the bacteria were plated on to BCYE plates and incubated for 37°C for 3 days.

### RNA extraction, cDNA synthesis, and qRT-PCR

RNA was extracted using Isolate II RNA Mini Kit (Bioline) as per manufacturer’s instructions. Equal amount of RNA was converted into cDNA using Superscript II Reverse Transcriptase (Invitrogen) with random hexamer primers following the manufacturer’s protocol unless otherwise specified. qRT-PCR was performed in 10 μL reactions in triplicate using Power SYBR® Green Master Mix (Thermo Fisher) according to the manufacturer’s protocol. In each 10 μL reaction, 0.5 μM primers and 1 μL of cDNA were used. QRT-PCR was then performed using QuantStudio 6 Flex Real-Time PCR System (Thermo Fisher). The relative mRNA level of each gene of interest was measured and normalized to the level of housekeeping gene *ACTB* using the delta-delta CT (∆∆CT) method, before normalizing the expression to the uninfected control.

### cDNA library preparation and multiplex RNA sequencing

RNA was extracted using Isolate II RNA Mini Kit (Bioline) as per manufacturer’s instructions. Four repeats were prepared, and the RNA integrity was examined using Agilent Bioanalyzer 2100. Only repeats with RNA integrity number (RIN) above 8.0 were used for the RNA sequencing experiment. The cDNA libraries for RNA sequencing were generated from 20 ng of RNA using an in-house multiplex RNA-seq method, where sample-specific indices were incorporated during oligo-d(T) priming of first strand cDNA (MHTP Medical Genomics Facility). Sequencing was performed on NextSeq2000 (Illumina) using P3 50 cycle kit. Parsing, base calling, adaptor trimming, and de-multiplexing were done using Dragen BCLConvert 3.7.4. The reads were then uploaded into the Galaxy platform for further analysis [86]. After checking for the absence of any adaptors and low-quality reads using FastQC [87], reads were aligned to the *Homo sapiens* genome (GrCh38) using RNA-STAR [88]. UMI de-duplication was done using UMI-tools deduplicate [89], and aligned reads were counted using featureCounts [90].

The subsequent analyses were performed in R (v.4.3.1). Genes with low expression (less than 10 counts in one sample, or less than 1 count per million across three samples) were filtered out. Data was normalized using the Trimmed Mean of M-values (TMM) method from the edgeR package, then transformed with voom and a linear model fit using lmFit from the limma package [91–94]. Statistical significance was then computed using TREAT with a fold-change threshold of 1.5 [95]. Log2 count per million (CPM) values relative to the average of the uninfected sample log2 CPM were used to plot the heatmaps. CAMERA gene set testing was performed to identify the enrichment of gene sets from the Reactome collection obtained from the Broad’s Institute Molecular Signature Database (MSigDB) [57,59,60]. Gene set heat maps were plotted using the log2 fold change compared to wild-type infected cells. The threshold for statistical significance was a false discovery rate (FDR)-adjusted p-value < 0.05.

### CRISPR knock-out in THP-1 cells

CRISPR mutagenesis was performed using paired guide RNAs cloned into pX461 (Addgene #48140) [96]. pX461 encoding gRNAs which targeted sense and antisense strands within 100 bp of a locus were mixed 1:1 and transfected into THP-1 monocytes using Lipofectamine LTX (ThermoFisher). GFP-positive cells were single cell sorted using a BD FACS Aria Fusion at the Monash Health Translational Precinct (MHTP) FlowCore and cultivated in RPMI + 10% FCS/Penicillin/Streptomycin. Individual clones were genotyped with PCR using primers which flank the CRISPR targeting site, and subsequently PCR products were Sanger sequenced to confirm exact mutations (or lack thereof). The expression of HSF1 was then confirmed using Western Blot with anti-HSF1 antibody (CST #4356, 1:1000).

### Cytotoxicity assay

Cell death was measured using the Promega CytoTox Non-Radioactive Assay according to the manufacturer’s protocol. Supernatant was collected from each well and spun down to remove dead cells and debris and 50 μL of the clarified supernatant was used in the assay

### Anti-HA, -TEM1, -Hsp90, -Hsc70 and -puromycin immunoblotting

Expression of HA-LegK4 in *L. pneumophila* culture was determined as follows. Δ*legK4* carrying either p4HA, p4HA-*legK4*, or p4HA-*legK4*_*D271A*_ was cultured for 18 h in ACES at 37˚C with agitation. Expression of HA-LegK4 was then induced by adding 1 mM IPTG for 3 h. After adjusting to an OD_600_ of 1, 0.5 mL of the culture was spun down and lysed in LDS sample buffer. Immunoblot was performed using anti-HA antibody (BioLegend Cat. 901514). Expression of TEM1-LPW_04851 in *L. pneumophila* culture was similarly determined by inducing with 1 mM IPTG for overnight and an immunoblot was performed using anti-TEM1 antibodies (QED Bioscience, Cat. 15720). For detection of heat shock proteins in *L. pneumophila* infected cells, differentiated THP-1 macrophages were infected with *L. pneumophila* for 8 h or 18 h, then lysed using KalB lysis buffer. Protein concentrations were determined using BCA assay, and 500 microgram was loaded onto 4–12% Bis-Tris Nupage gel. Immunoblot was performed using anti-Hsc70 (1:1000, Proteintech, Cat. 106541–1-AP), -Hsp90 (1:1000, Proteintech, Cat. 13171–1-AP), or -actin (1:5000, Sigma Cat. A5441). For puromycin experiments, differentiated THP-1 macrophages were infected with *L. pneumophila* for 8 h or 18 h, then labelled with 10 ug/mL puromycin (Gibco) for 15 minutes before lysis using KalB lysis buffer. Immunoblot was then performed using anti-puromycin (1:1000, Kerafast, Cat. EQ0001) or anti-actin (1:5000, Sigma Cat. A5441) antibodies.

### TEM1 translocation assay

To test for TEM1-LPW_04851 translocation, THP-1 monolayers were infected with *L. pneumophila* 130b or *DdotA* expressing TEM1-LPW_04851 (test) or TEM1- RalF (positive control) at an MOI of 40 for 1 h [53]. THP-1 cells were loaded with CCF2-AM (Invitrogen Cat#K1032) and incubated for an additional 90 min at room temperature prior to measuring fluorescence emission at 450 nm (blue) and 520 nm (green) in a CLARIOstar microplate reader (BMG Labtech). Translocation efficiency was expressed as the emission ratio of 450 nm/520 nm.

### Structural prediction of LPW_04851/Lpg0404

The structural model of LPW_04851 was constructed using the I-Tasser Server [97] for Protein Structure & Function Predictions. Analysis of the LPW_04851 structural model in comparison to *E. coli* GadC and structural figures were performed and generated using PyMOL (The PyMOL Molecular Graphics System, Version 3.0 Schrodiinger, LLC).

### Data submission and access

RNAseq data is available in the BioStudies database [98] (http://www.ebi.ac.uk/biostudies) under accession number E-MTAB-16655.

## Supporting information

S1 Fig Transcriptomic response of THP-1 macrophages during infection with wild-type *L. pneumophila* and mutant strains. A. Heat map of Hallmark gene sets that were differentially expressed during infection with different *Legionella* strains at an MOI of 10 compared to uninfected control. Gene set analysis was performed using CAMERA. Colour gradient indicates the average log2 fold change of the gene set, whereas numbers indicate the − log_10_(p-value). * indicates *p* value between 0.01 to 0.05. Scale was truncated to ±2. B. Heat map of Reactome gene sets that were differentially expressed during infection with different *Legionella* strains at an MOI of 10 compared to uninfected control. Gene set analysis was performed using CAMERA. Colour gradient indicates the log2 fold change of the gene set, whereas numbers indicate the − log_10_(p-value * indicates *p* value between 0.01 to 0.05. Scale was truncated to ±2.
(TIF)

S2 Fig Impact of *L. pneumophila* on the heat shock transcriptional response. A. Top gene sets upregulated by ∆E infection compared to wild-type *L. pneumophila* infection at an MOI of 10. Bars represent –log10 of FDR-adjusted p-value. B. Expression of *HSPB1* and *CRYAB*. Normalised log2 counts per million of the HSPB1 (upper panel) and CRYAB (lower panel) transcripts in THP1 macrophages after 18h of infection with different *L. pneumophila* mutant strains. Each dot represents one repeat. C. Percentage cell cytotoxicity in THP-1 cells during infection with wild-type *L. pneumophila* or mutant strains over 48h. Cell death assay was performed on supernatants during intracellular replication assay following infection with an MOI of 5. Bars represent the ratio of absorbance at 490 nm (A_490_) between each sample and the lysis control (100%) at each time point. Statistical significance was determined using unpaired t-test (* p < 0.05, ** p < 0.01), ns, results were not significantly different. D. Percentage cell cytotoxicity in *HSF1 + /+* or *HSF1-/-* THP-1 cells during infection with wild-type *L. pneumophila* or mutant strains infected at an MOI of 5 over 48 h. Bars represent the ratio of absorbance at 490 nm (A_490_) between each sample and the lysis control (100%) at each time point. Two independent guide RNAs (gRNAs) were used to generate the CRISPR KO cell lines, and at least one clone from each gRNA was used. Data from all clones of each genotype was pooled and represented as the mean of 4 replicates ± SEM. Statistical significance was determined using Mann-Whitney U (* p < 0.05, ** p < 0.01, *** p < 0.001, ****p < 0.0001). ns, results were not significantly different.
(TIF)

S3 Fig Impact of *L. pneumophila* on the heat shock translational response A. Levels of Hsc70 and Hsp90 heat shock proteins in THP-1 macrophages infected with derivatives of *L. pneumophila* 130b. Differentiated THP-1 macrophages were infected with *L. pneumophila* for 8 h or 18 h, then lysed using KalB lysis buffer. Immunoblot was performed using anti Hsc70, Hsp90, or Actin as a loading control. Image is representative of three independent repeats B. Protein synthesis in THP-1 macrophages infected with derivatives of *L. pneumophila* 130b. Differentiated THP-1 macrophages were infected at an MOI of 10. with *L. pneumophila* for 8 h (left panel) or 18 h (right panel) then labelled with 1 ug/mL puromycin for 15 minutes before lysis using KalB lysis buffer. Immunoblot was performed using anti puromycin or actin (loading controls) antibodies. Image is representative of three independent repeats.
(TIF)

S1 Table Comparison of the proteins in 130b strain to the reference Philadelphia strain. (XLSX)

S2 Table Single-nucleotide polymorphisms (SNPs) found in the genomic mutants. (DOCX)

S3 Table List of differentially expressed genes across all comparisons. (XLSX)

S4 Table Gene set analysis of enriched gene sets in the Reactome collection. (XLSX)

S5 Table List of genes downregulated during ∆S infection that is regulated by HSF1. (XLSX)

S6 Table List of bacterial strains used in this study. (DOCX)

S7 Table List of plasmids used in this study. (DOCX)

S8 Table List of oligonucleotides used in this study. (DOCX)
