# Supplementary material for: Multi-Dot/Icm effector mutants reveal distinct host responses in Legionella infected macrophages
Source: PLoS Pathog. 2026 Oct 22;22(10):e1014083. doi: 10.1371/journal.ppat.1014083 (PMC13712724; doi:10.1371/journal.ppat.1014083)
Supplement: Supplementary file 1 [file ppat.1014083.s005.docx]

## **Table S2. List of SNPs in the *L. pneumophila* genomic mutant strains**

| **Strain** | **SNPs** |
| --- | --- |
| ∆A | Intergenic region |
| ∆B | No SNP |
| ∆C | Deletion of T at position 76 in *lpw_31381* (phosphoenolpyruvate protein phosphotransferase *ptsP*), causing premature stop codon |
| ∆D | No SNP |
| ∆E | SNP in *lpw_20211* (major outer membrane protein), changing Thr2Ala |
| ∆F | No SNP |
| ∆G | Deletion of T in *lpw_28981* (Response regulator *letA*), causing a premature stop codon |
| ∆H | SNPs in *lvh1D4*, changing Ala68Val |
|  | Intergenic region |
|  | SNP in *lpw_31381*(phosphoenolpyruvate protein phosphotransferase *ptsP*), changing Glu480Ala |
| ∆I | Deletion of T at position 76 in *lpw_31381* (phosphoenolpyruvate protein phosphotransferase ptsP), causing premature stop codon |
| ∆O | SNP in *lpw_31381* (phosphoenolpyruvate protein phosphotransferase ptsP), changing Ser705Ala |
| ∆P | Intergenic region |
| ∆R | SNP in *lpw_24701* (hypothetical protein), causing the deletion of amino acids Arg186-188 |
| ∆S | Intergenic region |
| ∆FGHI | No SNP |
