# Supplementary material for: Multi-Dot/Icm effector mutants reveal distinct host responses in Legionella infected macrophages
Source: PLoS Pathog. 2026 Oct 22;22(10):e1014083. doi: 10.1371/journal.ppat.1014083 (PMC13712724; doi:10.1371/journal.ppat.1014083)
Supplement: Supplementary file 2 [file ppat.1014083.s009.docx]

## Table S6. List of bacterial strains used in this study

| **Strains** | **Characteristics** | **Sources/References** |
| --- | --- | --- |
| *E. coli*  XL1-Blue | *supE44 hsdR17 recA1 endA1 gyrA46 thi relA1 lac- F’ [proAB+ lacIq lacZM15 Tn10 (TetR )]* | Novagen |
| *E. coli*  PIR2 | *F-∆lac169 rpoS(Am) robA1 creC510  hsdR514 endA recA1 uidA(∆MluI)::pir* | Invitrogen |
| *L. pneumophila* 130b (ATCC BAA-744) | Clinical isolate | [1] |
| ∆*dotA* | 130b ∆*dotA* deletion mutant | [2] |
| ∆A | 130b ∆A deletion mutant | This study |
| ∆B | 130b ∆B deletion mutant | This study |
| ∆C | 130b ∆C deletion mutant | This study |
| ∆D | 130b ∆D deletion mutant | This study |
| ∆E | 130b ∆E deletion mutant | This study |
| ∆F | 130b ∆F deletion mutant | This study |
| ∆G | 130b ∆G deletion mutant | This study |
| ∆H | 130b ∆H deletion mutant | This study |
| ∆I | 130b ∆I deletion mutant | This study |
| ∆O | 130b ∆O deletion mutant | This study |
| ∆P | 130b ∆P deletion mutant | This study |
| ∆R | 130b ∆R deletion mutant | This study |
| ∆S | 130b ∆S deletion mutant | This study |
| ∆FGHI | 130b ∆FGHI deletion mutant | This study |
| ∆*legK4* | 130b ∆*legK4* deletion mutant | This study |
| ∆*lpw_02971* | 130b ∆*lpw_02971* deletion mutant | This study |
| ∆*lpw_02981* | 130b ∆*lpw_02981* deletion mutant | This study |

## References

1.	Edelstein PH. Control of *Legionella* in hospitals. J Hosp Infect. 1986;8(2):109-15. Epub 1986/09/01. doi: 10.1016/0195-6701(86)90037-x. PubMed PMID: 2876026.

2.	Riedmaier P, Sansom FM, Sofian T, Beddoe T, Schuelein R, Newton HJ, et al. Multiple ecto-nucleoside triphosphate diphosphohydrolases facilitate intracellular replication of *Legionella pneumophila.* Biochem J. 2014;462(2):279-89. Epub 2014/06/25. doi: 10.1042/BJ20130923. PubMed PMID: 24957128.
