# Supplementary material for: Multi-Dot/Icm effector mutants reveal distinct host responses in Legionella infected macrophages
Source: PLoS Pathog. 2026 Oct 22;22(10):e1014083. doi: 10.1371/journal.ppat.1014083 (PMC13712724; doi:10.1371/journal.ppat.1014083)
Supplement: Supplementary file 3 [file ppat.1014083.s010.docx]

## Table S7. List of plasmids used in this study

| **Plasmids** | **Characteristics** | **Sources/References** |
| --- | --- | --- |
| pSR47s | *sacB* based suicide vector for mutagenesis | [1] |
| pSR47s:∆A | Suicide vector to delete genomic region A | This study |
| pSR47s:∆B | Suicide vector to delete genomic region B | This study |
| pSR47s:∆C | Suicide vector to delete genomic region C | This study |
| pSR47s:∆D | Suicide vector to delete genomic region D | This study |
| pSR47s:∆E | Suicide vector to delete genomic region E | This study |
| pSR47s:∆F | Suicide vector to delete genomic region F | This study |
| pSR47s:∆G | Suicide vector to delete genomic region G | This study |
| pSR47s:∆H | Suicide vector to delete genomic region H | This study |
| pSR47s:∆I | Suicide vector to delete genomic region I | This study |
| pSR47s:∆O | Suicide vector to delete genomic region O | This study |
| pSR47s:∆P | Suicide vector to delete genomic region P | This study |
| pSR47s:∆R | Suicide vector to delete genomic region R | This study |
| pSR47s:∆S | Suicide vector to delete genomic region S | This study |
| pSR47s:∆*legK4* | Suicide vector to delete *legK4* | This study |
| pSR47s:∆*lpw_02971* | Suicide vector to delete *lpw_02971* | This study |
| pSR47s:∆*lpw_02981* | Suicide vector to delete *lpw_02981* | This study |
| pMMB207c_4xHA | N-terminal 4xHA expression vector | [2] |
| pMMB207c_4xHA:*legK4* | 130b *legK4* in pMMB207c_4xHA | This study |
| pMMB207c_4xHA:*legK4* | 130b *legK4* in pMMB207c_4xHA | This study |
| pTEM1-RalF | pXDC61 plasmid with *ralF* CDS | [3] |
| pTEM1-LPW_04851 | pXDC61 plasmid with *lpw_04851* CDS | This study |

## References

1.	Merriam JJ, Mathur R, Maxfield-Boumil R, Isberg RR. Analysis of the *Legionella pneumophila* *fliI* gene: intracellular growth of a defined mutant defective for flagellum biosynthesis. Infect Immun. 1997;65(6):2497-501. doi: 10.1128/iai.65.6.2497-2501.1997.

2.	Schroeder GN, Aurass P, Oates CV, Tate EW, Hartland EL, Flieger A, et al. *Legionella pneumophila* Effector LpdA Is a Palmitoylated Phospholipase D Virulence Factor. Infect Immun. 2015;83(10):3989-4002. Epub 20150727. doi: 10.1128/IAI.00785-15.

3.	Riedmaier P, Sansom FM, Sofian T, Beddoe T, Schuelein R, Newton HJ, et al. Multiple ecto-nucleoside triphosphate diphosphohydrolases facilitate intracellular replication of *Legionella* *pneumophila*. Biochem J. 2014;462(2):279-89. doi: 10.1042/BJ20130923.
