# Supplementary material for: Multi-Dot/Icm effector mutants reveal distinct host responses in Legionella infected macrophages
Source: PLoS Pathog. 2026 Oct 22;22(10):e1014083. doi: 10.1371/journal.ppat.1014083 (PMC13712724; doi:10.1371/journal.ppat.1014083)
Supplement: Supplementary file 4 [file ppat.1014083.s011.docx]

## **Table S8. List of oligonucleotide sequences**

| **Name** | **Sequence (5’-3’)** | **Purpose** |
| --- | --- | --- |
| pSR47s F | AGCTATGACCATGATTACGC | F- sequence pSR47s |
| pSR47s R | GTGAACGGCAGGTATATGT | R- sequence pSR47s |
| pMMB207c F | TTGACAATTAATCATCGGC | F- sequence pMMB207c-HAx4 |
| pMMB207c R | AGGCAAATTCTGTTTTATC | R- sequence pMMB207c-HAx4 |
| A5 F | GGACTAGTCGACACTCAAACCTCCACCC | F- amplify 5' flanking region of genomic region A. Contains SpeI site. |
| A5 R | AATAGAAGCCCCTTACCTCACAA | R- amplify 5' flanking region of genomic region A |
| A3 F | TTTTGTGAGGTAAGGGGCTTCTATTAAATGCCCCGCTTTCTGA | F- amplify 3' flanking region of genomic region A |
| A3 R | TTGCGGCCGCTTTTATCGGTGCTGTAATCTTGG | R- amplify 3' flanking region of genomic region A. Contains NotI site. |
| A_upstream_ | AAATCTGACCAATAGCCCAAGC | F- confirm the deletion of genomic region A |
| A_downstream_ | TTTGACGAAAAGGGAAACTGG | R- confirm the deletion of genomic region A |
| B5 F | CGAATTCCTGCAGCCCGGGGGATCCTGTCGGTTTCATTCAAATCCTC | F- amplify 5' flanking region of genomic region B. Contains BamHI site. |
| B5 R | TTCACCATATCCTGGCTCCAC | R- amplify 5' flanking region of genomic region B |
| B3 F | TGAGGTGGAGCCAGGATATGGTGAATGGTAATGACTCGCTCTGGG | F- amplify 3' flanking region of genomic region B |
| B3 R | CTAAAGGGAACAAAAGCTGGAGCTCCATGCTTTAGTTGCATAGGTTGTAT | R- amplify 3' flanking region of genomic region B. Contains SacI site. |
| B seq | GAAGTTGGTATGCGGGGTGC | Sequence pSR47s-ΔB |
| B_upstream_ | CTCGGTCAATATCGCTCATAGG | F- confirm the deletion of genomic region B |
| B_downstream_ | CATCGCCCTTGTATCGTTTG | R- confirm the deletion of genomic region B |
| C5 F | TTGCGGCCGCTGCTCGCAGCGCATCA | F- amplify 5' flanking region of genomic region C. Contains NotI site. |
| C5 R | TCCTCGCAAAACCACCCA | R- amplify 5' flanking region of genomic region C |
| C3 F | TATGCATTGGGTGGTTTTGCGAGGAGTGAAAACAATAATTGCTTGAAACC | F- amplify 3' flanking region of genomic region C |
| C3 R | CGGAGCTCTCCCAGATAATAGCCGAGTGC | R- amplify 3' flanking region of genomic region C. Contains SacI site. |
| C seq | TTGCGGAACGAACCA | Sequence pSR47s-ΔC |
| C_upstream_ | GTGTCAGGACGGCATAGGG | F- confirm the deletion of genomic region C |
| C_downstream_ | TCATCGCCTGCTTATGCTTG | R- confirm the deletion of genomic region C |
| D5 F | CGGGATCCCTGCTTTAGCGGGGAACC | F- amplify 5' flanking region of genomic region D. Contains BamHI site. |
| D5 R | AGAATGTGATAGGACGGTAGAAAAT | R- amplify 5' flanking region of genomic region D |
| D3 F | ATTTTCTACCGTCCTATCACATTCTAACAAGCGATTCGACTGATAAAA | F- amplify 3' flanking region of genomic region D |
| D3 R | TTGCGGCCGCCTGAAGAGGTAGATGCTGGTCC | R- amplify 3' flanking region of genomic region D. Contains NotI site. |
| D seq | CAGCCATGTTGGGTGAC | Sequence pSR47s-ΔD |
| D_upstream_ | CTGCTTTAGCGGGGAACC | F- confirm the deletion of genomic region D |
| D_downstream_ | TGCGTGCCTTATGATTCGTT | R- confirm the deletion of genomic region D |
| E5 F | GAGGGATCCGACCCTGTTGGCAGCTATC | F- amplify 5' flanking region of genomic region E. Contains BamHI site. |
| E5 R | TATCCCTACCTCTTGTGGAG | R- amplify 5' flanking region of genomic region E |
| E3 F | CTCCACAAGAGGTAGGGATAATGGCAGACAAATGTTAAGGAA | F- amplify 3' flanking region of genomic region E |
| E3 R | AGCGGCCGCCGGTTGGAGTAGTTTGGACA | R- amplify 3' flanking region of genomic region E. Contains NotI site. |
| E seq | AGCGGGTCCATATAAAATAGC | Sequence pSR47s-ΔE |
| E_upstream_ | GAACACGCGCTTGTAAGAAAA | F- confirm the deletion of genomic region E |
| E_downstream_ | AAATTGCAAAGATAAGATTGTACT | R- confirm the deletion of genomic region E |
| F5 F | CGAATTCCTGCAGCCCGGGGGATCCCAGTTTGCGGCCTCCACT | F- amplify 5' flanking region of genomic region F. Contains BamHI site. |
| F5 R | AGCAGCCTGTCGTCTATTATTTTAC | R- amplify 5' flanking region of genomic region F |
| F3 F | GTAAAATAATAGACGACAGGCTGCTTTGGCGGAAGTTGATGTCG | F- amplify 3' flanking region of genomic region F |
| F3 R | CTAAAGGGAACAAAAGCTGGAGCTCTGGCTTTCTCCACCAGGCT | R- amplify 3' flanking region of genomic region F. Contains SacI site. |
| F seq | CAAGGTCGAGGCAGAAAATG | Sequence pSR47s-ΔF |
| F_upstream_ | ATACGCCCTGATATGGGAAAT | F- confirm the deletion of genomic region F |
| F_downstream_ | GGCAAGAATAGTTCCTGTGCC | R- confirm the deletion of genomic region F |
| G5 F | CGGCGGCCGCCCCTGGCAATGTCACCTACC | F- amplify 5' flanking region of genomic region G. Contains NotI site. |
| G5 R | TACCTCCTGATTGTGGTTTTGTTT | R- amplify 5' flanking region of genomic region G |
| G3 F | AAAACAAAACCACAATCAGGAGGTAATCCACTTCCCTATGTCATGATTT | F- amplify 3' flanking region of genomic region G |
| G3 R | CGCGAGCTCGGCGTAAGGAAACGACAGG | R- amplify 3' flanking region of genomic region G. Contains SacI site. |
| G seq | TTGAAGTCACATTTGCATATCC | Sequence pSR47s- ΔG |
| G_upstream_ | TATCGATGAAAAGCACAGGAG | F- confirm the deletion of genomic region G |
| G_downstream_ | AATTCCATCCATTTTGGCGATA | R- confirm the deletion of genomic region G |
| H5 F | CGGGATCCGGAAAAGGAGCATCAAAACAGT | F- amplify 5' flanking region of genomic region H. Contains BamHI site. |
| H5 R | ATTAAGGAAAACCGAGGCAAA | R- amplify 5' flanking region of genomic region H |
| H3 F | ATTTTTTGCCTCGGTTTTCCTTAATTAACAACTGCCCTCATTGCTTT | F- amplify 3' flanking region of genomic region H |
| H3 R | CGGCGGCCGCCCATAGGTGCTTGCGCTACA | R- amplify 3' flanking region of genomic region H. Contains NotI site. |
| H seq | GCATGCCATCAAAATCCAATT | Sequence pSR47s-ΔH |
| H_upstream_ | GAGCTATATTTGCCCTTGGTA | F- confirm the deletion of genomic region H |
| H_downstream_ | ATCAGACTTGGTTGAAATTGTG | R- confirm the deletion of genomic region H |
| I5 F | CGGGATCCAATGATTGTCACTTGCGATGG | F- amplify 5' flanking region of genomic region I. Contains BamHI site. |
| I5 R | CGATTTGAGAATTATTTGAGGAGC | R- amplify 5' flanking region of genomic region I |
| I3 F | AGCTCCTCAAATAATTCTCAAATCGTATTGCTTTTCCTCCTTGGTTT | F- amplify 3' flanking region of genomic region I |
| I3 R | CGGCGGCCGCTCTTGCTGCTTTGGCTGTATC | R- amplify 3' flanking region of genomic region I. Contains NotI site. |
| I seq | TATCACGAAATGAGCAGACC | Sequence pSR47s-ΔI |
| I_upstream_ | AAAGAATAATTGGTTCGGCGA | F- confirm the deletion of genomic region I |
| I_downstream_ | ATTTCTCGCTCTCATTTGCAA | R- confirm the deletion of genomic region I |
| O5 F | TCGAATTCCTGCAGCCCGGGGGATCCCTTGGCAAGCATCCCTTATT | F- amplify 5' flanking region of genomic region O. Contains BamHI site. |
| O5 R | CTTGCGCATCACTTACGAAAACGGCAGCTGGTACATTTTT | R – amplify 5’ flanking region of genomic region O. |
| O3 F | AAAAATGTACCAGCTGCCGTTTTCGTAAGTGATGCGCAAG | F- amplify 3' flanking region of genomic region O. |
| O3 R | GAGCTCCACCGCGGTGGCGGCCGCAATCCGGGGATACCAATAGC | R- amplify 3' flanking region of genomic region O. Contains NotI site. |
| O seq | GAAACGGCAGAACAGCTGGAG | Sequence pSR47s-ΔO |
| O_upstream_ | CCTGCTAGAACGAAGCTTAATAAGCC | F- confirm the deletion of genomic region O |
| O_downstream_ | GCATGCCCCAATGGAGACC | R- confirm the deletion of genomic region O |
| P5 F | TCGAATTCCTGCAGCCCGGGGGATCCTTGGCCTATTCCGCTTACAC | F- amplify 5' flanking region of genomic region P. Contains BamHI site. |
| P5 R | CAAACAACTCTAGAATGACCGGTGCGTATCCATTTTTGAGGA | R- amplify 5' flanking region of genomic region P. |
| P3 F | TCCTCAAAAATGGATACGCACCGGTCATTCTAGAGTTGTTTG | F- amplify 3' flanking region of genomic region P. |
| P3 R | GAGCTCCACCGCGGTGGCGGCCGCGGCCGATTATCTTCCAGTGA | R- amplify 3' flanking region of genomic region P. Contains NotI site. |
| P seq | TGTAATCCCGGGTGTCGTTGA | Sequence pSR47s-ΔP |
| P_upstream_ | ATTGGTGACCGACCAGAGAAGC | F- confirm the deletion of genomic region P |
| P_downstream_ | GGTGCATACATCCTATAATCAAGCGG | R- confirm the deletion of genomic region P |
| R5 F | TCGAATTCCTGCAGCCCGGGGGATCCCGCATTTTTGACGGTAGGAT | F- amplify 5' flanking region of genomic region R. Contains BamHI site. |
| R5 R | AAAAGCGAAACGCTCTCTCACGGTATTTACCGCCACTTGT | R- amplify 5' flanking region of genomic region R. |
| R3 F | ACAAGTGGCGGTAAATACCGTGAGAGAGCGTTTCGCTTTT | F- amplify 3' flanking region of genomic region R. |
| R3 R | GAGCTCCACCGCGGTGGCGGCCGCTATGGCCCATGTCAGACAAA | R- amplify 3' flanking region of genomic region R. Contains NotI site. |
| R seq | GTTGCCAGTCAACTCCCATCC | Sequence pSR47s-ΔR |
| R_upstream_ | CCATGGCAATTTTCTTTTGTTGATCGC | F- confirm the deletion of genomic region R |
| R_downstream_ | CGTATGCGGTGAAGGACAACC | R- confirm the deletion of genomic region R |
| S5 F | AGCGGCCGCGTTCATTCCCCAAGCGTTTA | F- amplify 5' flanking region of genomic region S. Contains NotI site. |
| S5 R | AACAGAATATATTGCCCTCTGAAGAAGCAAAATAGCTGCTCCCAAG | R- amplify 5' flanking region of genomic region S |
| S3 F | TTCTTCAGAGGGCAATATATTCTGTT | F- amplify 3' flanking region of genomic region S |
| S3 R | TCGGAGCTCGCTATGAACCACAGGCCATT | R- amplify 3' flanking region of genomic region S. Contains SacI site. |
| S seq | CATTCAATCGGATTCAGGTAA | Sequence pSR47s-ΔS |
| S_upstream_ | TGTTACTCTACATGAAGACAA | F- confirm the deletion of genomic region S |
| S_downstream_ | CCATCATGAAATTGTAATTCCAC | R- confirm the deletion of genomic region S |
| dLegK4 5 F | GTCGGATCCCGCCTTCGTTATGCCTACAT | F- amplify 5' flanking region of *legK4*. Contains BamHI site. |
| dLegK4 5 R | AAGGAACCTCACATATTTACAAACTTG | R- amplify 5' flanking region of *legK4* |
| dLegK4 3 F | CAAGTTTGTAAATATGTGAGGTTCCTTCCTCAAGGAAGGCGTTTTATG | F- amplify 3' flanking region of *legK4* |
| dLegK4 3 R | TAGGAGCTCCTTCTTTCAAATCGGGGTGA | R- amplify 3' flanking region of *legK4*. Contains SacI site. |
| dLegK4_upstream_ | TTTTGGGTGAAAAGGCATGT | F- confirm the deletion of *legK4* |
| dLegK4_downstream_ | TGATTGGCTTCTTGTGCTTG | R- confirm the deletion of *legK4* |
| 2971 5 F | GTCGGATCCCTGAAAACGGACTGCCCTTA | F- amplify 5' flanking region of *lpw_02971*. Contains BamHI site. |
| 2971 5 R | AAACTATTTCCCTTTTTCCTTTACAACAAAACGCCTTCCTTGAGGTTA | R- amplify 5' flanking region of *lpw_02971.* |
| 2971 3 F | GTTGTAAAGGAAAAAGGGAAATAGTTT | F- amplify 3' flanking region of *lpw_02971.* |
| 2971 3 R | AGCGGCCGCCTCGTTTGGCTTCTTTTTGC | R- amplify 3' flanking region of *lpw_02971.* Contains NotI site. |
| 2971_upstream_ | TGGTCAATCCTGGTATGCTG | F- confirm the deletion of *lpw_02971.* |
| 2971_downstream_ | CTGGGCTTCTGATTTTGAGC | R- confirm the deletion of *lpw_02971.* |
| 2981 5 F | GTCGGATCCCAAGCACAAGAAGCCAATCA | F- amplify 5' flanking region of *lpw_02981*. Contains BamHI site. |
| 2981 5 R | ACAGAATATATTGCCCTCTGAAGAAAAACTATTTCCCTTTTTCCTTTACAAC | R- amplify 5' flanking region of *lpw_02981.* |
| 2981 3 F | TTCTTCAGAGGGCAATATATTCTGT | F- amplify 3' flanking region of *lpw_02981.* |
| 2981 3 R | TAGGAGCTCCTGACGCCAGTTCCATTTTT | R- amplify 3' flanking region of *lpw_02981*. Contains SacI site. |
| 2981_upstream_ | CTGGATCATTCAGCGCATAG | F- confirm the deletion of *lpw_02981.* |
| 2981_downstream_ | CAATTCATCAACCCTGGACA | R- confirm the deletion of *lpw_02981.* |
| LegK4 F | GAGGTACCATGAAATTGCTTCGGTTTCATGAATT | F- amplify *legK4*. Contains KpnI site. |
| LegK4 R | GTCGGATCCTTAATATGGCAAAATGATGACGTTGCGG | R- amplify *legK4*. Contains BamHI site. |
| D271A F | CAGTGTCATTTAGTTGCTTTCGGCAGCAGTTCTTC | F- mutagenesis of residue D271A of *legK4*. |
| D271A R | GAAGAACTGCTGCCGAAAGCAACTAAATGACACTG | R- mutagenesis of residue D271A of *legK4*. |
| HSPA6 F | CGTGCCCGCCTATTTCAATG | F- qPCR for human *HSPA6*. |
| HSPA6 R | AAAAATGAGCACGTTGCGCT | R- qPCR for human *HSPA6*. |
| HSPA4L F | TGTACCCCGGCCTGTATATCA | F- qPCR for human *HSPA4L*. |
| HSPA4L R | ACGTTCGTGACTATCTGGCTC | R- qPCR for human *HSPA4L*. |
| DNAJA4 F | CAGGTGAGGTGATAAAGCACG | F- qPCR for human *DNAJA4*. |
| DNAJA4 R | TCTGTCGAGGAGGGAGTAAAG | R- qPCR for human *DNAJA4*. |
| SERPINH1 F | TGCTAGTCAACGCCATGTTCT | F- qPCR for human *SERPINH1*. |
| SERPINH1 R | ATAGGACCGAGTCACCATGAA | R- qPCR for human *SERPINH1*. |
| BAG3 F | GGAGATCAAGATCGACCCGC | F- qPCR for human *BAG3*. |
| BAG3 R | AGAGGATGGAGTCTCCTTGGG | R- qPCR for human *BAG3*. |
| ACTB F | GCGAGAAGATGACCCAGATC | F- qPCR for human *ACTB*. |
| ACTB R | CCAGTGGTACGGCCAGAGG | R- qPCR for human *ACTB*. |
| HSF1 1A S | CACCGAAAGTGGTCCACATCGAGC | F- Sense oligonucleotide to make gRNA 1 to knock out human *HSF1*. |
| HSF1 1A A | AAACGCTCGATGTGGACCACTTTC | F- Antisense oligonucleotide to make gRNA 1 to knock out human *HSF1*. |
| HSF1 1B S | CACCGAAGCCATCTGCACATAAC | R- Sense oligonucleotide to make gRNA 1 to knock out human *HSF1*. |
| HSF1 1B A | AAACGTTATGTGCAGATGGCTTC | R- Antisense oligonucleotide to make gRNA 1 to knock out human *HSF1*. |
| HSF1 2A S | CACCGCACCCATGCTTCCTGCG | F- Sense oligonucleotide to make gRNA 2 to knock out human *HSF1*. |
| HSF1 2A A | AAACCGCAGGAAGCATGGGTGC | F- Antisense oligonucleotide to make gRNA 2 to knock out human *HSF1*. |
| HSF1 2B A | CACCGCTCGATGTGGACCACTTTC | R- Sense oligonucleotide to make gRNA 2 to knock out human *HSF1*. |
| HSF1 2B S | AAACGAAAGTGGTCCACATCGAGC | R- Antisense oligonucleotide to make gRNA 2 to knock out human *HSF1*. |
| HSF1 F | CAGTGGCCGCTCTTCAGG | F – genotyping human *HSF1* deletion. |
| HSF1 R | GGCACTCACACTGGTCACTT | R – genotyping human *HSF1* deletion. |
| TEM1- LPW_04851 F | CGGGGTACCATGAGCAATAAAAAACATTCTCTCACT | To create a translational fusion of LPW_04851with TEM1 in pXDC61 for translocation assay |
| TEM1- LPW_04851 R | TCTAGATTATACAGCAGGTTCCAAATCCG | To create a translational fusion of LPW_04851with TEM1 in pXDC61 for translocation assay |
